# Metabolite-Resolved Validation of Bacterial Organophosphate Transformation in Soil: A Critical Review of Selected Organophosphate Case Studies

**DOI:** 10.3390/microorganisms14091974

**Published:** 2026-09-07

**Authors:** Lazzat Asylbekkyzy, Meruyert O. Bauenova, Assemgul K. Sadvakasova, Dariga K. Kirbayeva, Bekzhan D. Kossalbayev, Fiaz Ahmad, Dilnaz E. Zaletova

**Affiliations:** 1 Department of Biotechnology, Faculty of Biology and Biotechnology, Al-Farabi Kazakh National University, Al-Farabi 71, Almaty 050038, Kazakhstan; assylbekkyzy_lazzat3@live.kaznu.kz (L.A.); bauyenova.meruyert@kaznu.edu.kz (M.O.B.); kk.dariga@gmail.com (D.K.K.); zaletova_dilnaz2@live.kaznu.kz (D.E.Z.); 2 Department of Chemical and Biochemical Engineering, Satbayev University, 22 Satpaev Street, Almaty 050013, Kazakhstan; 3 Ecology Research Institute, Khoja Akhmet Yassawi International Kazakh-Turkish University, Turkistan 161200, Kazakhstan; 4 Key Laboratory for Space Bioscience & Biotechnology, School of Life Science and Technology, Northwestern Polytechnical University, Chang’an Campus, Xi’an 710072, China; fiaz.a@mail.nwpu.edu.cn

**Keywords:** organophosphate pesticides, bacterial transformation, soil bioremediation, transformation products, catabolic enzymes, microbial consortia

## Abstract

This structured critical narrative review evaluates the bacterial transformation of six selected organophosphate pesticides—chlorpyrifos, methyl parathion, parathion, diazinon, malathion, and phosalone—in relation to chemical, mechanistic, and environmental evidence. Peer-reviewed literature was searched in Scopus and PubMed for the period from 1 January 2020 to 26 August 2026 and was supplemented by backward-reference screening and forward citation tracking of foundational studies. Evidence was assessed across eight domains spanning association, tolerance, controlled dissipation, product-resolved transformation, mechanistic attribution, non-sterile-soil validation, detoxification or ecological recovery, and field readiness. The selected compounds reveal substantial differences in pathway resolution: chlorpyrifos has the most developed parent–product evidence base, methyl parathion and parathion are comparatively well characterized at the hydrolytic and enzyme levels, diazinon and malathion retain important downstream uncertainties, and phosalone remains an unresolved evidence-gap comparator. The synthesis shows that credible soil bioremediation requires convergence among parent–product kinetics, catalytic attribution, transformation-product turnover, performance beyond natural attenuation, toxicity reduction, and recovery of relevant soil or plant functions.

## 1. Introduction

Chemical pesticides remain important for rapid crop protection, but their residues can move beyond target sites and persist after use. A global assessment found that 64% of agricultural land is at risk of pollution by more than one pesticide and 31% is exposed to high pollution risk [1], while residues have also been detected in organically managed soils decades after conversion from conventional agriculture [2]. Such legacy contamination is biologically relevant because residue profiles correlate with reduced bacterial diversity and *nifH* abundance [3], whereas greater pesticide diversity is associated with impairment of several nutrient-cycling functions [4]. Pesticide residues in agricultural environments may also reach edible crop tissues, extending the consequences of environmental contamination to dietary exposure. Residue occurrence in root, tuber, and bulb vegetables further indicates the relevance of this pathway to food safety and associated human-health risks.

Organophosphate pesticides (OPPs) require compound-specific evaluation. Differences in phosphoester or phosphorothioate bonding, leaving groups, ester architecture, lipophilicity, and hydrolytic stability influence sorption, enzyme recognition, and the products formed during transformation [5]. Consequently, disappearance of a parent OPP may indicate detoxification, incomplete conversion to a persistent or biologically active product, or merely non-biological loss; mechanisms cannot be transferred among OPPs solely because they share a phosphorus-containing functional group.

A major weakness in the literature is the frequent use of parent-compound decline as proof of biodegradation. Abiotic hydrolysis or oxidation, volatilization, sorption, sequestration, and incomplete analytical recovery can generate comparable declines [6,7], while tentative mass-spectral annotations are not equivalent to product identities confirmed with authentic standards or orthogonal evidence [8]. Product-resolved studies illustrate different evidential levels: *Arthrobacter* sp. HM01 linked chlorpyrifos loss to 3,5,6-trichloro-2-pyridinol (TCP) and 2,6-dihydroxypyridine [9]; an indigenous consortium enhanced malathion transformation relative to its constituent isolates, although substantial dissipation also occurred in uninoculated soil [10]; and diazinon-degrading strain X1 produced 2-isopropyl-6-methyl-4-pyrimidinol (IMP) without metabolizing it further [11]. Tolerance, controlled dissipation, transformation-product formation, downstream-product turnover, detoxification, and mineralization must therefore be treated as distinct claims.

Taxonomic or predicted functional data likewise cannot assign catalysis. PICRUSt2 and other 16S rRNA gene-based approaches infer gene content from reference genomes and are constrained by strain-level variation and incomplete representation of accessory catabolic genes [12,13]. Active mechanism requires correspondence among gene expression, protein or enzyme activity, and time-resolved parent–product kinetics. Translation from culture to soil adds constraints imposed by pore-scale heterogeneity [14], residue ageing and remobilization of bound residues [15], and the size and resupply of the labile contaminant pool [16].

Remediation should therefore be judged by chemical evidence together with recovery of relevant soil functions. Soil health depends on the capacity of a living system to sustain organisms and ecosystem services [17,18], and pesticide residues can alter microbial and faunal diversity and nutrient cycling [19]. These issues are especially important in saline and semi-arid soils, where salinity modifies pesticide fate and microbial activity [20]. In the Almaty Region, residue composition was associated with bacterial community turnover and predicted functions, although such associations do not identify the organisms responsible for transformation [21].

Previous reviews cover natural attenuation, biostimulation, bioaugmentation, and chlorpyrifos-remediation mechanisms [22,23], but chemical fate, catalytic attribution, toxicity, and soil recovery are often assessed separately. The present review addresses this fragmentation through a metabolite-resolved evidence chain extending from controlled parent-compound depletion to product formation and turnover, gene-enzyme-reaction attribution, performance beyond natural attenuation in non-sterile soil, toxicity reduction, and functional recovery.

Accordingly, this review does not attempt an exhaustive survey of all organophosphate pesticides. Instead, six compounds—chlorpyrifos, methyl parathion, parathion, diazinon, malathion, and phosalone—were selected as contrasting case studies spanning different chemical structures, transformation products, catalytic mechanisms, and levels of environmental validation. Chlorpyrifos represents the most extensively studied parent–product system; methyl parathion and parathion illustrate PNP-forming hydrolytic pathways; diazinon represents incomplete turnover of the heterocyclic product IMP; malathion illustrates branched ester-cleavage routes; and phosalone is retained as an unresolved evidence-gap comparator. Within this defined scope, the review applies a common metabolite-resolved framework to determine which combinations of chemical, mechanistic, and environmental evidence are sufficient to support claims of bacterial transformation, detoxification, and soil-remediation competence. Studies involving non-organophosphate pesticides or general methodological approaches are used only as explicitly labelled comparator evidence and do not contribute to compound-specific soil-remediation maturity.

## 2. Literature Search and Critical Evidence-Synthesis Strategy

This review followed a structured critical narrative approach supported by reproducible database searching, predefined eligibility criteria, and study-level evidence classification. A systematic review or quantitative meta-analysis was not considered appropriate because the available literature differed substantially in pesticide identity and concentration, bacterial system, experimental matrix, analytical coverage, control design, and environmental endpoints. The search therefore aimed not only to identify relevant studies, but also to determine the type and level of experimental support that each study could legitimately provide.

Peer-reviewed publications were identified through Scopus and PubMed, supplemented by backward-reference screening and forward citation tracking [24]. The original contemporary search covered the period from 1 January 2020 to 30 June 2026. During revision, both database searches were repeated and updated on 26 August 2026; consequently, the final retrieval record reported here extends through that date. Earlier publications were not included in the contemporary database window, but citation tracking allowed their retention when they contained foundational information that could not be replaced by more recent work, such as direct enzyme characterization, transformation-product identification, radiolabelled fate analysis, or historically established pathway evidence. These earlier publications were treated separately as foundational evidence and were not used to support claims concerning the absence of contemporary compound-specific validation. The database queries combined general terms for organophosphate pesticides with terms related to bacterial transformation, biodegradation, bioremediation, transformation products, catabolic genes, and enzyme activity, while chlorpyrifos, methyl parathion, parathion, diazinon, malathion, and phosalone were also included as individual case-compound terms. Complete search strings, search dates, publication windows, database-specific retrieval numbers, eligibility criteria, and reviewer roles are reported in Supplementary Table S1.

The updated searches yielded 682 records from Scopus and 254 from PubMed, giving 936 records before deduplication. DOI-based matching, followed by bibliographic reconciliation where required, removed 220 duplicate records and left 716 unique publications for title-and-abstract screening. Of these, 434 did not meet the predefined criteria, whereas 282 were shortlisted for detailed eligibility and evidentiary assessment. Application of the evidence criteria resulted in the retention of 41 database-derived publications. Backward and forward citation tracking, together with targeted selection of foundational, methodological, and contextual literature, contributed a further 40 publications, producing a final evidence-synthesis corpus of 81 studies. Each retained publication was then classified at study level. Studies contributing directly to compound-specific conclusions were distinguished from methodological or contextual comparators, which were retained for interpretation but did not increase compound-specific evidence maturity. The complete study-level classification is presented in Supplementary Table S2.

Direct compound-specific evidence required investigation of a bacterial strain, consortium, community, or catalytic system in relation to an organophosphate pesticide or a defined transformation product, together with at least one experimentally interpretable chemical, molecular, toxicological, ecological, or environmental endpoint. Studies involving non-organophosphate pesticides, general microbiome or analytical methodology, and other systems used primarily for methodological comparison were retained only when they provided transferable interpretive information. These studies were explicitly classified as comparator evidence and were excluded from compound-specific soil-remediation maturity assignments. Engineered or culture-based studies involving a focal organophosphate pesticide could contribute to compound-specific mechanistic or transformation evidence, but did not by themselves constitute non-sterile-soil or field validation.

To avoid subjective labels such as “strong” or “weak” evidence, all retained studies were evaluated against explicit operational criteria. Association denoted a reproducible link between pesticide exposure and a bacterial taxon, community, abundance pattern, network property, or biological function without direct demonstration of transformation. Tolerance required survival or growth under a defined pesticide exposure relative to an appropriate baseline or control and was not taken as evidence of catalytic conversion. Controlled dissipation required time-resolved quantitative loss of the parent compound in comparison with an abiotic, uninoculated, or otherwise appropriate control, whereas product-resolved transformation required detection of one or more transformation products associated temporally or chemically with parent-compound loss. Mechanistic attribution was reserved for studies that experimentally linked a gene, protein, enzyme, or catalytic function to the measured chemical reaction; gene presence, genome annotation, docking, predicted catalytic function, or proteomic association alone were treated as mechanistic potential rather than causal proof. Non-sterile-soil validation required maintenance of the relevant transformation phenotype in soil containing resident microbiota and natural attenuation processes. Detoxification or ecological recovery required a directly measured reduction in toxicity or recovery of a defined soil or plant function, while field readiness required environmentally realistic field-scale efficacy together with evidence of persistence or functional maintenance, transformation-product control, and adequate biosafety information. Experimental scale was recorded separately for each study as defined liquid culture or enzyme system, freshly spiked sterile soil, freshly spiked non-sterile soil, aged or historically contaminated non-sterile soil, greenhouse experiment, mesocosm or constructed wetland, or true field validation.

Analytical confidence was evaluated independently from the evidence domains. Transformation-product evidence was classified from P0 to P4. P0 indicated that no transformation-product evidence was reported. P1 denoted indirect pathway assignment without direct analytical identification of the proposed product. P2 indicated tentative analytical identification based on a single analytical platform, retention behaviour, molecular-ion information, or library matching without diagnostic fragmentation or authentic-standard confirmation. P3 required stronger analytical support from diagnostic fragmentation, tandem mass spectrometry, accurate mass, or independent orthogonal evidence. P4 was reserved for transformation products confirmed against an authentic analytical standard, including retention-time and spectral agreement.

Gene and enzyme evidence was classified in parallel from G0 to G3. G0 indicated the absence of relevant mechanistic evidence. G1 represented gene detection, genome annotation, or identification of a predicted candidate enzyme. G2 denoted expression, proteomic, or enzyme-activity evidence associated with transformation but lacking causal validation, whereas G3 required direct functional evidence from a purified or recombinant enzyme, catalytic-site mutation, knockout/complementation, or an equivalent causal experiment. A study could satisfy more than one evidence domain when each criterion was independently met. Supplementary Table S2 records the experimental matrix, pesticide concentration, bacterial system, controls, analytical method, product-confirmation level, gene or enzyme evidence, toxicity or functional endpoints, experimental scale, and resulting evidence assignment for each retained study.

Mineralization was treated separately from these domains because parent-compound disappearance or transformation-product turnover does not by itself demonstrate complete degradation. Mineralization therefore required direct carbon-fate, radiolabel, or equivalent mass-balance evidence attributable to the pesticide. Similarly, CO_2_ production without demonstrated pesticide-derived carbon attribution was not considered sufficient.

L.A. conducted the primary literature screening and evidence extraction using the predefined criteria, after which D.E.Z. independently verified study eligibility and evidence-domain assignments. Any differences in eligibility or classification were discussed and resolved by consensus, and A.K.S. reviewed the final classification. This procedure was applied consistently to the study-level evidence matrix presented in Supplementary Table S2.

Finally, conclusions concerning evidence gaps were restricted to the documented search corpus rather than interpreted as proof of biological absence. Phosalone was therefore retained as an unresolved evidence-gap comparator because no study within the predefined contemporary corpus used in this synthesis established a continuous experimental chain linking controlled bacterial phosalone depletion with analytically supported transformation products, direct catalytic attribution, and validation in non-sterile soil. The available corpus therefore does not yet establish an integrated validation chain for phosalone, but this gap should not be interpreted as demonstrating that bacterial transformation of the compound is biologically impossible.

## 3. Compound-Specific Transformation Pathways and Validation Priorities

Contemporary bacterial studies indicate that chlorpyrifos transformation is strain-dependent, with differences in removal kinetics, metabolic requirements, and adaptation to pesticide exposure [25,26]. Product-resolved studies further separate initial chlorpyrifos hydrolysis from subsequent TCP turnover, showing that these stages may proceed through distinct strain-specific routes [27,28,29]. Comparable hydrolysis-dominated patterns are reported for methyl parathion, parathion, and diazinon, where formation of *p*-nitrophenol (PNP) or IMP can be distinguished analytically from their downstream metabolism [11,30,31,32,33,34,35]. Malathion follows a less uniform pattern because cleavage at multiple ester bonds generates alternative product sequences rather than a single consistently reproduced pathway [10,36,37,38]. Phosalone remains less resolved: within the predefined 2020–2026 corpus, no eligible primary study combined controlled bacterial depletion, analytically confirmed transformation products, catalytic attribution, and non-sterile-soil validation. Contemporary syntheses likewise discuss bacterial pesticide degradation broadly without providing a comparably integrated strain–product–enzyme pathway for phosalone [39]. Across these compounds, pathway maturity is therefore best evaluated from the continuity of evidence linking parent loss, product formation, downstream turnover, and reaction-specific catalytic support [39].

### 3.1. Hydrolysis-Dominated Pathways

Among the organophosphate pesticides considered in this review, chlorpyrifos provides the most suitable mechanistic model for illustrating how soil-controlled bioavailability, initial enzymatic cleavage, and downstream microbial metabolism jointly determine bacterial transformation [29]. The pathway begins with access by microbial cells or extracellular catalytic functions to the bioavailable pesticide fraction, followed by hydrolytic cleavage to TCP and diethyl thiophosphate. Subsequent TCP turnover depends on strain-specific metabolic capacity, expression of downstream catalytic functions, and, in mixed systems, functional cooperation among microbial populations.

**Figure 1:**
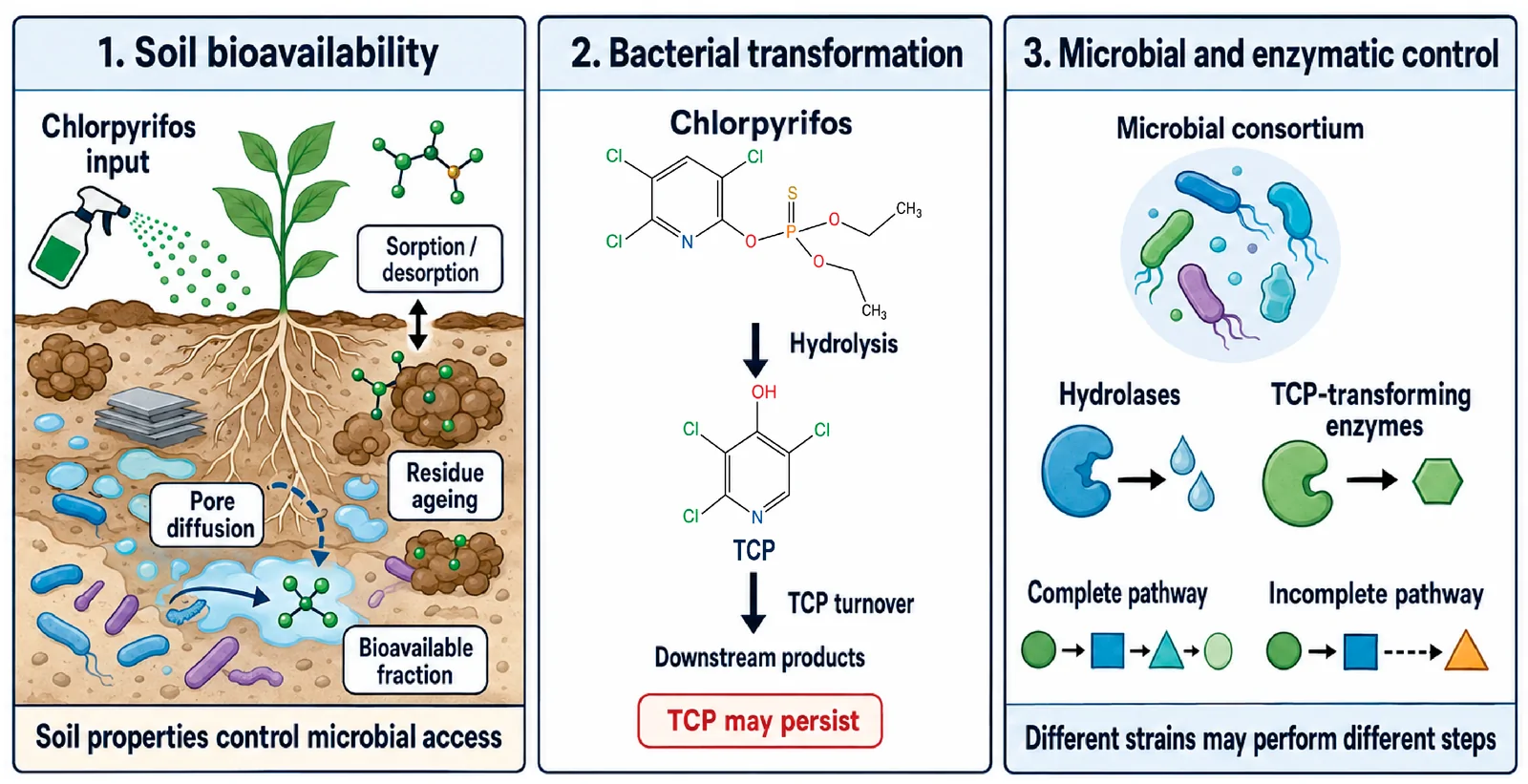
Soil bioavailability and bacterial transformation of chlorpyrifos.

Figure 1 therefore uses chlorpyrifos as a comparatively well-resolved example linking soil exposure, parent-compound hydrolysis, and subsequent product turnover. *Arthrobacter* sp. HM01 generated TCP and subsequently 2,6-dihydroxypyridine, providing a chemically resolved connection between chlorpyrifos depletion and downstream metabolism [27]. *Bacillus thuringiensis* MB497 transformed both chlorpyrifos and TCP through a different product sequence, indicating that TCP turnover is not conserved across bacterial strains [28]. Initial chlorpyrifos hydrolysis and subsequent TCP metabolism should therefore be considered distinct functional stages that may involve different enzymes or cooperating microbial populations [29].

The pathway proposed for *Bacillus* sp. H27 further extended this resolution through detection of 14 transformation products assigned to three possible routes. More than 80% of chlorpyrifos was removed under the tested conditions, while cellular-fraction experiments localized most measured activity to intracellular components. Whole-genome analysis additionally identified transport-associated functions, esterases, phosphatases, and stress-response traits potentially associated with the observed phenotype. In this case, residue kinetics, product profiling, and cellular-enzyme fractionation provide complementary mechanistic support, whereas genome annotation alone indicates functional potential. However, quantitative mineralization requires direct evidence for pesticide-derived carbon or labelled atoms in inorganic end products.

Parallel chlorpyrifos and TCP kinetics provide the chemical basis for resolving initial hydrolysis and downstream pathway progression [29]. TCP formation establishes the primary cleavage step, whereas its subsequent decline, together with the temporally consistent appearance of additional analytically supported intermediates, allows reconstruction of later reactions.

A comparable hydrolysis-to-product sequence occurs with methyl parathion, which releases *p*-nitrophenol (PNP) [33]. During transformation by *Burkholderia zhejiangensis* CEIB S4-3, methyl-parathion depletion was accompanied by PNP removal and proteomic responses associated with hydroquinone- and hydroxyquinol-related metabolism, oxidative-stress protection, and xenobiotic processing [30]. The correspondence between chemical turnover and proteomic response provides molecular support for the observed transformation phenotype.

Direct biochemical attribution of the initial reaction has also been achieved [31]. A methyl parathion hydrolase from *Azohydromonas australica* was cloned, heterologously expressed, and characterized against methyl parathion and several related organophosphorus substrates, establishing a direct gene–enzyme–reaction relationship for primary cleavage without resolving subsequent PNP metabolism.

Engineered systems have reconstructed the connection between methyl-parathion hydrolysis and downstream aromatic metabolism [32,33]. Co-expression of an organophosphorus hydrolase and the *pnpA–pnpE* pathway enabled engineered *Escherichia coli* to couple parent-compound cleavage with conversion of the released aromatic product under defined conditions [32]. A complementary engineered strain transformed PNP through a hydroquinone-associated route toward β-ketoadipate and central cellular metabolism [33]. These systems establish the biochemical feasibility of pathway completion, but not stable expression, competitive persistence, or ecological compatibility in non-sterile soil.

Parathion also generates PNP following hydrolytic cleavage, although its evidence base remains less integrated than that of methyl parathion [34]. Recombinant OpdA and OpdE from *Leuconostoc mesenteroides* WCP307 hydrolysed parathion and related organophosphorus substrates, while modification of essential catalytic residues reduced or abolished activity. This directly supports the involvement of these hydrolases in the initial reaction but does not establish subsequent PNP turnover. The more extensively reconstructed methyl-parathion pathway therefore cannot be extrapolated to parathion solely because both compounds release PNP.

Diazinon follows an analogous hydrolytic pattern but releases the heterocyclic product IMP. In a defined bacterial system, 200 µM diazinon was converted to IMP within 6 h, while transcriptional analysis, heterologous expression, and enzyme assays identified OpdB as the principal hydrolase [11]. IMP persisted during bacterial incubation and required subsequent photochemical treatment for further removal, demonstrating that the bacterial process resolved the initial hydrolytic step but not downstream IMP turnover.

A different transformation sequence was reported for *Sphingobium* sp. DI-6, which removed approximately 92% of diazinon from mineral medium within 60 h and generated additional strain-specific products [35]. The contrast between these systems indicates that downstream diazinon metabolism depends on strain-level metabolic capacity. Simultaneous measurement of diazinon, IMP, and additional confirmed intermediates is therefore required to resolve pathway progression beyond initial cleavage.

Across chlorpyrifos, methyl parathion, parathion, and diazinon, hydrolysis products such as TCP, PNP, and IMP provide chemically specific markers of the first transformation step, whereas downstream pathway resolution depends on subsequent product turnover and corresponding catalytic evidence [35]. This parent-to-product framework becomes less linear for malathion because multiple ester bonds permit alternative transformation routes [10].

### 3.2. Variable and Incompletely Resolved Pathways: Malathion and Phosalone

Malathion transformation is characterized by product diversity arising from cleavage of different ester bonds under strain- and matrix-dependent conditions [38]. An indigenous bacterial consortium tested in amended non-sterile soil produced greater malathion removal than its individual members and generated multiple transformation products [10]. Dissipation also occurred in uninoculated soil, indicating that the contribution of bioaugmentation must be evaluated against an appropriate natural-attenuation baseline.

Culture-based studies further demonstrate that malathion does not follow a single conserved bacterial sequence [36,37,38]. *Ochrobactrum* sp. M1D generated methyl phosphate, diethyl maleate, and diethyl 2-mercaptosuccinate and removed 100 mg L^−1^ malathion within 12 days under mineral-medium conditions [36]. *Micrococcus aloeverae* MAGK3 produced a different chromatographic product pattern, while optimization of culture conditions increased malathion removal to approximately 87% within 38 h [37]. These contrasting profiles support alternative ester-cleavage routes rather than one universally applicable pathway.

Further product-resolved evidence was obtained for *Pseudidiomarina homiensis* FG2 and *Pseudidiomarina* sp. CB1, which transformed 500 mg L^−1^ malathion within 36 h under defined culture conditions [38]. Malathion monoacid and diacid derivatives were identified as major intermediates, while genome analysis revealed several candidate carboxylesterase genes [38]. The correspondence between ester-cleavage chemistry, detected products, and genomic potential supports the proposed mechanism, but direct catalytic attribution remains unresolved. Functional validation of the implicated carboxylesterases would require expression analysis, purified-enzyme assays, inhibition, mutation, or gene-disruption experiments.

Taken together, the available evidence supports several strain-dependent malathion transformation routes rather than a single canonical sequence [10]. The main unresolved questions concern assignment of individual products to causal enzymes and confirmation that downstream turnover remains active in non-sterile soil [38].

Phosalone occupies a different evidential position. No eligible primary study within the predefined contemporary corpus established an integrated bacterial pathway linking controlled parent-compound depletion with confirmed transformation products, active catalytic functions, and performance in non-sterile soil. Contemporary reviews discuss bacterial degradation mechanisms for several pesticides but do not provide a comparably validated, strain-resolved phosalone pathway [39]. This gap reflects limitations of the available evidence rather than proof that bacterial transformation of phosalone is chemically impossible, and extrapolation from chlorpyrifos, parathion, or other organophosphorothioates would therefore be unsupported.

Resolving the phosalone pathway will require controlled enrichment or isolation of competent strains, time-resolved measurements with abiotic and uninoculated controls, high-resolution mass-spectrometric product discovery followed by authentic-standard confirmation, and direct gene–enzyme–reaction attribution. Subsequent testing in aged non-sterile soil should determine whether product turnover, inoculant persistence, toxicity reduction, and relevant soil functions are maintained.

Across the selected case compounds, malathion therefore represents a pathway with multiple experimentally supported but incompletely attributed ester-cleavage routes, whereas phosalone remains an unresolved evidence-gap case lacking an integrated strain–product–enzyme validation chain (Table 1) [39].

**Table 1:** Compound-specific transformation markers, analytical priorities and principal validation gaps.

Pesticide	Principal Transformation Markers	Required Analytical Evidence	Current Evidence Status	Principal Validation Gap	References
Chlorpyrifos	TCP; 2,6-dihydroxypyridine and other strain-specific products	Parallel chlorpyrifos and TCP kinetics; time-resolved downstream-product turnover; analytical confirmation of major intermediates	Controlled dissipation and product-resolved transformation are demonstrated in defined bacterial systems; mechanistic attribution is available for selected enzyme systems.	Complete TCP turnover, direct mineralization evidence, product-specific toxicity assessment, and replicated validation in aged non-sterile soil	[25,26,27,28,29]
Methyl parathion	PNP; hydroquinone-, hydroxyquinol-, and β-ketoadipate-associated products	Separate methyl-parathion and PNP kinetics; direct hydrolase validation; downstream-pathway activity synchronized with chemical analysis	Controlled dissipation, product-resolved transformation, and mechanistic attribution are demonstrated in defined and engineered systems.	Stable operation of the complete pathway, PNP turnover, detoxification, and validation in aged non-sterile soil	[30,31,32,33]
Parathion	PNP; downstream aromatic products where experimentally resolved	Compound-specific parathion and PNP kinetics; direct enzyme analysis; analytical confirmation of downstream-product turnover	Mechanistic attribution is demonstrated for the initial hydrolytic reaction, whereas downstream product-resolved transformation remains incompletely established.	Downstream PNP turnover, detoxification, and validation of sustained transformation in non-sterile soil	[34]
Diazinon	IMP; strain-specific downstream products	Parallel diazinon and IMP kinetics; confirmation of additional intermediates; direct assessment of active hydrolase function	Controlled dissipation and product-resolved transformation are demonstrated, and the initial hydrolytic step is mechanistically resolved in selected systems; downstream outcomes remain strain-dependent.	Complete IMP turnover, product-specific toxicity assessment, and replicated non-sterile-soil validation	[11,35]
Malathion	Malathion monoacid and diacid derivatives; methyl phosphate; diethyl maleate; diethyl 2-mercaptosuccinate	Broad time-resolved product profiling; natural-attenuation controls; direct functional testing of candidate carboxylesterases	Controlled dissipation and product-resolved transformation are demonstrated through several strain- and matrix-dependent routes, including culture and soil systems.	Direct catalytic attribution of individual ester-cleavage reactions, complete product fate, toxicity reduction, and reproducibility in aged non-sterile soil	[10,36,37,38]
Phosalone	No strain-specific bacterial transformation marker established within the predefined contemporary evidence corpus	Controlled parent-compound kinetics; high-resolution product discovery; authentic-standard or equivalent product confirmation; direct gene–enzyme–reaction attribution	No study within the predefined contemporary corpus met the combined criteria for product-resolved bacterial transformation, mechanistic attribution, and non-sterile-soil validation.	Identification of competent bacterial strains, confirmed primary and downstream products, active catalytic functions, and validation in non-sterile soil	[39]

## 4. Translation of Liquid-Culture Performance to Soil Bioremediation

Compound-specific pathways are commonly resolved first in defined liquid media, where substrate exposure, nutrient composition, oxygen availability, and incubation conditions can be tightly controlled [40]. These systems establish catalytic competence but only partially predict performance in soil, where inoculant activity is constrained by establishment, competition, substrate accessibility, and interactions with the resident microbiome. Soil structure further partitions microorganisms, water, nutrients, and contaminants among pores with different connectivity and metabolic conditions [41]. Consequently, transformation observed in dissolved culture systems may decline after sorption, matrix retention, or spatial separation of competent degraders from accessible pesticide fractions [41,42].

### 4.1. Spatial Heterogeneity, Sorption and Residue Accessibility

Pore-scale heterogeneity creates distinct microbial habitats within the same bulk soil [41]. Communities occupying contrasting pore domains differ in composition and metabolic characteristics, and millimetre-scale aggregation of degrader populations can reduce encounters with pesticide molecules even when the required catabolic capacity is present [42]. Although the underlying spatial model was developed for 4-chloro-2-methylphenoxyacetic acid rather than an organophosphate pesticide, it illustrates a transferable constraint: catalytic potential may remain unexpressed when competent cells and accessible substrate are physically separated.

Sorption and desorption further regulate the fraction of pesticide available in soil solution [43,44]. Dimethoate retention varied among soils with different physicochemical properties, while clay-associated retention and desorption demonstrated that organic matter, mineral composition, and pesticide–surface interactions influence residue mobility and persistence. Transformation kinetics are therefore matrix-dependent. Comparative experiments involving four pesticides and five urban soils likewise showed pesticide–soil-specific degradation behaviour, indicating that rates obtained in one matrix cannot be generalized without considering texture, organic carbon, mineral composition, pH, and biological properties [45].

Contamination history introduces an additional constraint. Freshly spiked residues have had less time to interact with sorptive domains and may therefore remain more accessible than aged residues [44]. High removal in newly amended microcosms can consequently overestimate activity against legacy contamination. Evaluation under environmentally relevant conditions should distinguish freshly spiked from aged soil and, where possible, separate total extractable residues from the fraction accessible to microbial transformation.

Natural attenuation must also be quantified before the contribution of an introduced treatment can be interpreted [46]. Chlorpyrifos dissipation occurred in uninoculated horticultural-soil microcosms, confirming that indigenous microbial activity and matrix-dependent processes contribute to pesticide loss. The effect of bioaugmentation or biostimulation should therefore be assessed relative to an uninoculated non-sterile control rather than from the final concentration in the treated soil alone.

### 4.2. From Inoculation to Environmentally Relevant Validation

Once the natural-attenuation baseline is established, the intervention can be matched to the process limiting transformation in the target soil [47]. Combined bioaugmentation and biostimulation enhanced chlorpyrifos dissipation in laboratory soil microcosms, but endpoint removal alone could not distinguish direct activity of the introduced population from stimulation of indigenous degraders or interactions between the two. Inoculant tracking, transformation-product kinetics, and community responses are therefore required to resolve the biological contribution of the treatment.

*Serratia rubidaea* ABS 10 illustrates the distinction between enhanced dissipation and more complete chemical fate resolution [48]. The strain accelerated chlorpyrifos disappearance in sterile and non-sterile agricultural soil, but radiolabel evidence did not demonstrate extensive mineralization. Adsorption to biomass and formation of biogenic non-extractable residues remained plausible contributors to the measured decline [48]. The study therefore supports enhanced dissipation across soil matrices without establishing extensive mineralization.

Formulation and immobilization can improve transfer from culture to soil by protecting cells and increasing their retention near contaminated microsites [49]. A formulated preparation of *Dyadobacter jiangsuensis* strain 12,851 enhanced chlorpyrifos remediation in contaminated soil, while immobilization of *Bacillus* sp. H27 increased removal relative to the tested free-cell and carrier treatments [50]. However, carrier-assisted systems can also alter pesticide sorption and extractability independently of microbial metabolism. Carrier-only, inoculum-only, and combined treatments, together with time-resolved parent and product measurements, are therefore required to distinguish biological transformation from physicochemical retention [50].

Environmental complexity can be increased further by co-stressors that are absent from optimized culture systems [51]. Polylactic-acid microplastics altered chlorpyrifos degradation, TCP dynamics, phosphorus bioavailability, and phosphatase activity, with responses varying according to microplastic loading. Such interactions indicate that pesticide accessibility and microbial physiology may be modified simultaneously in soils affected by plastics or mixed contaminants.

Chemical dissipation should also be evaluated alongside functionally relevant ecological endpoints. Chlorpyrifos inhibited nitrogen fixation, rhizosphere colonization, extracellular polymeric substance production, and biofilm formation by *Pseudomonas stutzeri* A1501 in rice-vegetated soil, whereas total bacterial abundance did not reflect these functional impairments [52]. Ecological recovery therefore requires direct measurement of the soil or plant process expected to respond to remediation.

Ecotoxicological measurements provide an additional test of whether chemical treatment reduces biological risk [53]. A remediation system combining bacterial strains with cyclodextrin evaluated chlorpyrifos removal together with soil ecotoxicity. Because cyclodextrin also modifies pesticide availability, its physicochemical contribution must be distinguished from bacterial transformation. Chemical mobilization, microbial conversion, and toxicity reduction should therefore be treated as related but separate components of remediation performance.

Environmental validation can thus be viewed as a progressive increase in matrix complexity, from sterile and non-sterile soil to aged residues, co-contaminated systems, mesocosms, and field conditions [40,46,51,53]. Advancement across these levels is best supported when transformation-product turnover, inoculant or functional persistence, toxicity reduction, and recovery of a relevant soil or plant process are evaluated together with parent-compound depletion [48].

## 5. Evidence Framework for Classifying Bacterial Organophosphate-Transforming Systems

Translation from laboratory screening to environmental application requires classification that remains proportional to the experimental matrix, analytical resolution, mechanistic evidence, and biological endpoints examined [54,55]. Pesticide association, tolerance, controlled dissipation, product-resolved transformation, catalytic attribution, non-sterile-soil validation, and bioinoculant candidacy therefore represent distinct evidential stages rather than interchangeable indicators of degradation competence.

### 5.1. From Pesticide Association to Product-Resolved Transformation

Contaminated soils and enrichment cultures are useful discovery matrices because repeated pesticide exposure can enrich primary hydrolysers, downstream-product utilizers, cometabolic partners, and stress-tolerant populations. Growth in pesticide-amended medium, however, may also be supported by residual nutrients, formulation additives, carrier solvents, or metabolites released by other community members. Newly recovered organisms should therefore remain classified as pesticide-associated or pesticide-tolerant candidates until their activity is linked to controlled chemical measurements.

Progression beyond candidate status requires reproducible parent-compound depletion relative to appropriate controls. In soil, this includes an uninoculated natural-attenuation treatment, while killed-cell controls can help assess biomass-associated sorption where relevant. Analytical interpretation should also account for calibration, extraction recovery, matrix effects, detection limits, and temporal sampling [55]. Physiological measurements such as optical density or viable-cell counts can support interpretation but do not substitute for residue and transformation-product analysis.

*Bacillus* sp. UPMB10 illustrates the value and limitations of culture-level chemical evidence [56]. Under optimized mineral-medium conditions containing 4 g L^−1^ chlorpyrifos, the strain produced approximately 99% parent-compound depletion within 48 h, while GC–MS analysis yielded product assignments including mesitylene and dimethylmalonic acid. These observations support a product-informed culture-level transformation phenotype, but the high substrate concentration, defined liquid matrix, and analytical confidence of the reported product assignments limit extrapolation to aged non-sterile soil. Authentic-standard confirmation, diagnostic fragmentation or orthogonal analytical evidence, together with time-resolved product turnover, would be required to resolve the proposed pathway more rigorously.

Across organophosphate pesticides, product-resolved classification depends on temporal continuity between parent loss, appearance of a chemically plausible transformation product, and its subsequent decline or conversion. TCP, PNP, and IMP therefore provide compound-specific markers of hydrolytic transformation, whereas detoxification and mineralization require separate toxicological and atom-resolved evidence.

### 5.2. Strain Resolution and Causal Attribution of Catabolic Functions

The chemical transformation has been established; mechanistic analysis should determine which strain and catalytic function are responsible for the measured reaction [55]. Taxonomic identity and gene detection alone are insufficient because catabolic traits may be strain-specific, accessory, horizontally transferred, or conditionally expressed. Stronger attribution requires experimental linkage among the candidate gene or protein, substrate-specific catalytic activity, and the expected chemical reaction.

The *opdC* system from *Lactiplantibacillus plantarum* WCP931 provides a direct example [57]. The *opdC* gene was cloned and expressed heterologously; the approximately 31 kDa enzyme was characterized, and recombinant cells were transformed with chlorpyrifos under defined conditions. Replacement of the predicted catalytic Ser116 residue with alanine abolished activity toward the tested organophosphorus substrates, directly linking the encoded enzyme to the initial reaction. This establishes gene–enzyme attribution for primary cleavage, although downstream-product turnover and soil persistence remain unresolved.

Proteomic evidence occupies an intermediate position. Chlorpyrifos exposure induced extensive proteome remodelling in *Pseudomonas nitroreducens* AR-3, including proteins associated with transport, central metabolism, oxidative phosphorylation, and xenobiotic transformation [58]. A presumptive metallo-β-lactamase-fold hydrolase with similarity to an organophosphorus hydrolase was also identified. These synchronized molecular responses support mechanistic interpretation, but direct attribution of the candidate hydrolase would require heterologous expression or purification, substrate-specific enzyme assays, and functional perturbation. AR-3 is therefore more appropriately classified as a proteomically supported transforming system than as an enzyme-resolved pathway.

Causal attribution becomes more complex in consortia because different pathway stages may be distributed among multiple populations [59]. Consortium C3, composed of *Lactiplantibacillus plantarum*, *Lacticaseibacillus rhamnosus*, and *Bacillus shackletonii*, transformed chlorpyrifos, cypermethrin, and glyphosate under optimized culture conditions and removed approximately 94.93% of chlorpyrifos after 15 days. GC–MS analysis supported pathway proposals, but specific reactions could not be assigned to individual members, and performance across chemically distinct pesticides should not be interpreted as a single conserved catabolic pathway.

Member-specific pathway functions should therefore be resolved through reconstruction of defined consortia, selective omission and reintroduction of individual strains, and measurement of the chemical step affected by each manipulation. Strain-specific qPCR, transcript analysis, metaproteomics, stable-isotope tracing, or cell-resolved methods can further connect populations to particular stages. Until such evidence is obtained, consortium performance should be classified according to the demonstrated collective phenotype rather than presumed metabolic complementarity.

### 5.3. From Soil Validation to Bioinoculant Candidacy

Culture-level chemical and mechanistic evidence must ultimately be connected to performance in non-sterile soil [54,55]. Soil validation requires activity beyond natural attenuation, maintenance of the relevant transformation function under matrix constraints, and evidence that environmentally important transformation products do not simply accumulate.

*Enterobacter* sp. HSTU-ASh6 illustrates the need to separate evidence obtained in different experimental systems [60]. The strain transformed chlorpyrifos in culture, and GC–MS/MS analysis supported formation of TCP and an additional organophosphorus-containing product. Whole-genome analysis identified candidate pesticide-associated and plant-growth-promoting functions, while foliar application improved tomato growth under field conditions with reduced urea input. Because pesticide kinetics and transformation products were not measured in the field plant-growth experiment, these findings support product-resolved culture transformation and independently demonstrated plant-beneficial traits, but not field-validated chlorpyrifos remediation.

Greater environmental integration was achieved in a plant–microbe–biochar system combining *Klebsiella pneumoniae*C78, *Ricinus communis*, and biochar in chlorpyrifos-spiked soil [61]. Soil microcosm and pot experiments showed accelerated chlorpyrifos depletion relative to non-inoculated treatments, no accumulation of the targeted products TCP, DETP, or TMP under the tested conditions, and improvements in soil biological properties and plant-performance indicators. This supports classification as an integrated soil-tested remediation system, although the absence of targeted products alone does not establish quantitative mineralization, and biochar may independently influence sorption, extractability, and contaminant release.

Because C78 was identified as *K. pneumoniae*, strain-level biosafety remains essential before environmental deployment [62]. Species identity alone cannot exclude virulence determinants, antimicrobial-resistance genes, toxins, secretion systems, or transferable mobile elements. Whole-genome safety screening, phenotypic assessment, and evaluation of gene-transfer potential are therefore required before soil efficacy can be translated into bioinoculant candidacy.

Environmental validation should also distinguish persistence of the inoculated population from persistence of the required function. An introduced strain may decline while leaving a transient remediation effect, persist without maintaining catalytic activity, or alter the resident microbiome after pesticide concentrations have fallen. Functional persistence is therefore better defined as maintenance of the required transformation activity throughout the remediation period, with post-treatment monitoring used where prolonged ecological effects are relevant [54].

Within this framework, advancement from pesticide-associated candidate to environmentally credible bioinoculant requires progressively stronger chemical, mechanistic, soil-level, and biosafety evidence. The same logic can be applied across organophosphate pesticides, provided that transformation products, catalytic reactions, and environmental endpoints are defined separately for each target compound (Table 2).

**Table 2:** Evidence-based classification of representative bacterial organophosphate-transforming systems.

System	Demonstrated Evidence	Appropriate Classification	Evidence Still Required	Ref.
*Bacillus* sp. UPMB10	Chlorpyrifos depletion in mineral medium and GC–MS product assignments	Product-supported culture-level transformer	Authentic-standard product confirmation, time-resolved product turnover, enzyme attribution, non-sterile-soil validation and toxicity testing	[56]
OpdC system from Lactiplantibacillus plantarum WCP931	Gene cloning, heterologous expression, enzyme characterization and loss of activity after catalytic-site mutation	Direct gene–enzyme attribution for initial organophosphorus-pesticide cleavage	Complete product pathway, product-specific toxicity, persistence and soil performance	[57]
Pseudomonas nitroreducens AR-3	Chlorpyrifos transformation accompanied by proteome remodelling and identification of candidate catalytic proteins	Proteomically supported culture-level transforming system	Direct attribution of individual proteins, downstream-product turnover and non-sterile-soil validation	[58]
Consortium C3	Multi-pesticide depletion, genome-based member identification and GC–MS-informed pathway proposals	Product-informed multi-pesticide consortium	Member-specific pathway functions, reconstruction experiments, product confirmation, persistence and soil validation	[59]
*Enterobacter* sp. HSTU-ASh6	Chlorpyrifos transformation with product analysis, whole-genome characterization and separately demonstrated plant-growth promotion	Mechanistically informed culture-level transformer with plant-beneficial traits	Atom-resolved mineralization, active-enzyme attribution, pesticide measurements in the plant–soil treatment, biosafety and non-sterile-soil validation	[60]
Klebsiella pneumoniae C78–Ricinus communis–biochar system	Chlorpyrifos dissipation in soil and pot experiments, targeted product monitoring and plant/soil responses	Integrated soil-tested remediation system	Strain-level biosafety, comprehensive product and mass-balance analysis, inoculant persistence, product-specific toxicity and field replication	[61,62]

## 6. Microbiome-Level Evidence in Organophosphate-Contaminated Soils

The strain-level framework becomes environmentally relevant when bacterial transformation is considered within the resident soil microbiome. Field application of chlorpyrifos has been associated with changes in bacterial abundance, community composition, soil enzyme activities, and genes involved in carbon and nitrogen cycling, although the direction and magnitude of these responses varied across crop stages and functional endpoints [63,64]. Comparative field evidence further showed that chlorpyrifos, phosalone, and dimethoate produced distinct effects on basal and substrate-induced microbial respiration [65]. Broader pesticide studies likewise indicate that community restructuring depends on compound identity, exposure intensity, application regime, and soil properties rather than following a uniform pesticide-response pattern [66,67].

The soil microbiome therefore functions both as an ecological target of pesticide disturbance and as a potential source of organisms involved in parent-compound or transformation-product turnover. Exposure-enriched taxa may represent primary hydrolysers, downstream-product utilizers, stress-tolerant populations, or organisms responding indirectly to altered nutrient and carbon flows. Community-level observations are consequently most informative when interpreted together with compound-specific chemistry and functional measurements.

### 6.1. Community Restructuring and Ecological Interpretation

Taxonomic and biochemical responses to pesticide exposure do not necessarily change in parallel. Dimethachlor and linuron altered bacterial composition, microbial biomass, respiration, and enzyme activities, but the magnitude and temporal trajectories differed among compounds and concentrations [68]. Meta-analytic evidence likewise indicates that nutrient-cycling markers may respond consistently to pesticide pressure even when broad diversity indices remain comparatively stable [69]. Alpha diversity alone is therefore insufficient for assessing functional impairment or recovery.

Interpretation is further complicated by agricultural management. In field soils receiving pesticides and mineral fertilizers, sampling time and soil properties explained substantial variation in bacterial community structure, while treatment-associated shifts also involved taxa potentially carrying antibiotic-resistance determinants [70]. Although this does not establish direct selection of specific resistance genes by pesticides, it indicates that remediation-oriented microbiome assessment may need to include biosafety-related endpoints in addition to taxonomic diversity.

Greater interpretive value is obtained when community responses are measured together with pesticide chemistry and soil functions. Under aromatic-grass vegetation, chlorpyrifos dissipation was evaluated alongside TCP accumulation, soil-enzyme kinetics, root-associated effects, and bacterial-community responses, showing that accelerated parent loss could coexist with persistence of the hydrolytic product [71]. In a biochar-amended soil–plant system, chlorpyrifos and TCP measurements were integrated with microbial-community analysis, enzyme activity, and pesticide uptake, while biochar simultaneously modified contaminant availability and biological responses [72]. These studies demonstrate that microbiome restructuring is most informative when linked to parent–product kinetics and amendment-specific controls.

Evidence obtained from different experimental systems should nevertheless remain distinct. Dose-dependent soil-enzyme responses and culture-level chlorpyrifos transformation by *Pseudomonas citronellolis* CF3 provided complementary information, but the two observations were generated under different conditions and did not constitute a single soil-remediation pathway [73].

Microbiome statistics introduce an additional layer of uncertainty. Alpha-diversity estimates depend on sequencing depth, prevalence filtering, sample size, and metric selection, while beta-diversity analyses may reflect shifts in community centroid, within-group dispersion, or both [74]. Constrained ordination, distance-based tests, and component-based methods can also produce different representations of the same intervention effect [75]. Reporting should therefore include preprocessing decisions, dispersion assessment, effect sizes, and the biological rationale for the selected metrics.

Taxon-level differential-abundance analysis requires similar caution. Comparative evaluation of multiple methods revealed substantial disagreement in the taxa identified as significantly enriched or depleted [76]. Robust taxon-level conclusions should therefore consider prevalence, effect magnitude, multiple-testing correction, biological replication, and stability across defensible analytical approaches. Community-level evidence can characterize ecological disturbance and identify candidates for mechanistic investigation, but reaction-specific transformation still requires direct chemical and functional evidence.

### 6.2. From Taxonomic Profiles to Functional Evidence

The inferential distance increases when taxonomic profiles are converted into predicted metabolic functions. The accuracy of marker-gene-based functional inference depends on reference-genome coverage, phylogenetic proximity, marker resolution, and conservation of the predicted trait [77]. Comparative soil studies have shown that such tools can recover broad metabolic tendencies while underestimating functional diversity relative to shotgun metagenomics [78]. This limitation is particularly important for accessory or strain-variable catabolic traits.

Benchmarking of 16S rRNA gene-based metagenome prediction has shown that inferred functions may diverge from directly measured genomic content when target genes are unevenly distributed among closely related organisms [13]. Expansion of the PICRUSt2 reference database through PICRUSt2-SC improves genomic representation and average predictive performance, but does not establish that a predicted gene is present, expressed, or catalytically active in the environmental population [79]. Predicted xenobiotic-degradation functions should therefore remain classified as inferred potential.

Shotgun metagenomics provides a more direct measure of environmental gene content. Agricultural soils with prolonged pesticide exposure contained candidate taxa, catabolic functions, and putative degradation pathways that were not accessible through conventional amplicon profiling alone [80]. A separate long-term pesticide study similarly revealed changes in community structure, functional genes, and stress-associated microbial potential [81]. These approaches improve pathway resolution at the DNA level, but active pathway execution still requires temporal linkage to transcription, protein abundance, enzyme activity, and chemical transformation.

Long-term diazinon exposure illustrates how community and cultivation data can be integrated. Repeated treatment enriched *Actinomycetota* in soil microcosms, placed members of this phylum in central network positions, and yielded diazinon-transforming isolates, approximately 70% of which belonged to *Actinomycetota* [82]. This convergence provides a stronger basis for candidate selection than taxonomic enrichment alone, while still requiring isolate-level product analysis and mechanistic attribution.

Cultivation-dependent and cultivation-independent analyses of chlorpyrifos-associated microbiota also recovered overlapping but non-identical functional components [83]. Enrichment-community profiling identified populations not fully represented among cultivable isolates, while organophosphorus-hydrolase genes differed between the mixed enrichment and an individual strain. Isolation may therefore omit organisms involved in pathway completion, whereas community-level gene detection cannot assign a specific reaction to a particular population without population-resolved linkage.

A microbiome-informed workflow can consequently progress from amplicon profiling to shotgun metagenomics, cultivation, and activity-resolved approaches such as metatranscriptomics, metaproteomics, or enzyme assays. Its mechanistic value is greatest when these measurements are obtained from the same temporal experiment and synchronized with parent-pesticide and transformation-product kinetics.

### 6.3. Network Inference and Experimental Validation of Community Interactions

Microbial association networks identify taxa whose abundance patterns covary across samples, but network edges do not directly demonstrate competition, cooperation, or metabolite exchange. Shared responses to pesticide concentration, pH, nutrients, or sampling time may generate positive associations, while compositional effects can produce apparent negative relationships [84]. Network position should therefore be interpreted as hypothesis-generating rather than reaction-specific evidence.

Network topology is also sensitive to prevalence filtering, normalization, compositional correction, association method, and edge-selection thresholds. Comparative analyses have shown that different workflows can recover substantially different network structures from the same amplicon dataset [85]. Claims concerning complexity, modularity, robustness, or keystone status should therefore be accompanied by transparent analytical reporting and sensitivity assessment [84].

Topological importance also remains distinct from catalytic responsibility. Experimental manipulation of soil bacterial diversity demonstrated that specialized functions associated with particular taxa can contribute disproportionately to community resistance under environmental perturbation [86]. Such observations can prioritize candidate populations for further study, but highly connected taxa are not necessarily responsible for the chemically decisive step of organophosphate transformation.

This distinction is evident in chlorpyrifos-contaminated soil, where microbial inoculation altered community organization and several inferred network-stability characteristics [87]. These changes indicate treatment-associated restructuring of the resident microbiome but do not identify whether the inoculated population hydrolysed chlorpyrifos, transformed TCP, or indirectly supported indigenous degraders. Reaction-specific attribution therefore requires integration of network observations with chlorpyrifos and TCP kinetics, inoculant tracking, and measurements of active catabolic functions.

Network-derived hypotheses can be tested experimentally through controlled consortium reconstruction. Selective omission and reintroduction of individual members can determine whether a population is required for initial hydrolysis, downstream-product turnover, or primarily for community stability. Strain-specific qPCR can quantify persistence, while metatranscriptomics, metaproteomics, targeted enzyme assays, gene disruption, and stable-isotope approaches can connect populations to active pathway stages. Isotope incorporation should nevertheless be interpreted cautiously because cross-feeding may label downstream consumers that did not perform the initial reaction.

Microbiome recovery should finally be evaluated through restoration of an ecologically relevant process rather than exact return to the taxonomic composition of an uncontaminated reference soil. Functional redundancy may permit recovery of respiration, nutrient cycling, or plant performance despite persistent compositional differences, whereas limited taxonomic change may coexist with substantial functional impairment. Environmental validation should therefore integrate temporal community profiling with product-resolved chemistry and at least one directly measured soil or plant function.

Within this framework, microbiome analysis characterizes ecological disturbance, identifies candidate organisms and functions, tracks inoculant establishment, and generates experimentally testable hypotheses concerning community interactions. Its strongest contribution arises when taxonomic, molecular, biochemical, and chemical measurements are obtained from the same experimental system and converge temporally with transformation-product turnover and functional recovery [87].

## 7. Integrated Validation Framework for Translating Bacterial Organophosphate Transformation into Soil Remediation

**Figure 2:**
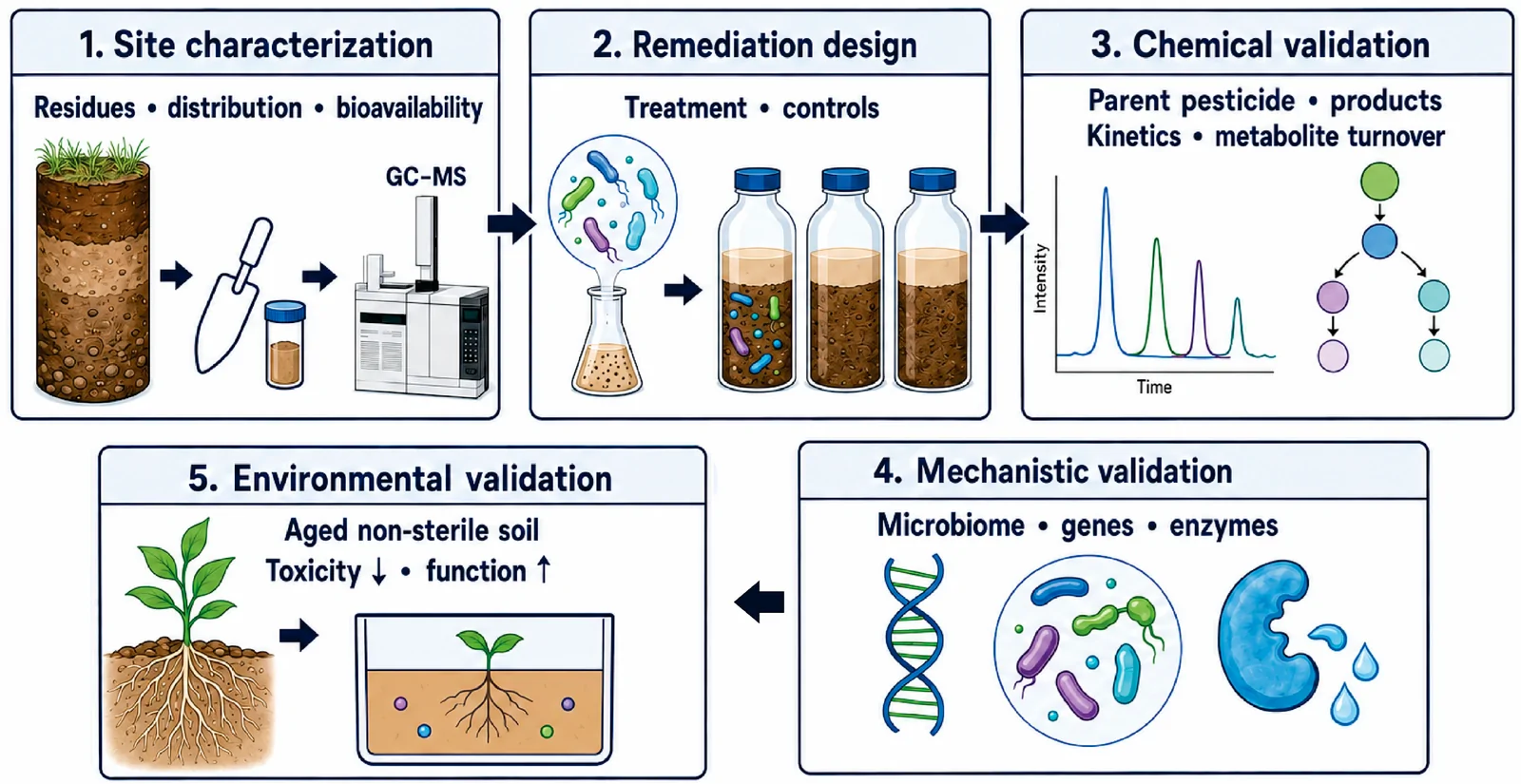
Stepwise validation framework for translating bacterial organophosphate transformation into soil remediation.

Microbiome-level evidence can identify pesticide-associated populations, candidate catabolic functions, and treatment-induced ecological responses, but its practical significance depends on connection to measurable chemical transformation. Translation into soil remediation therefore requires continuity among site characterization, intervention design, parent-compound and transformation-product kinetics, active catalytic functions, performance beyond natural attenuation, and recovery of biologically relevant endpoints. To our knowledge, no widely adopted framework currently integrates these evidence domains specifically for bacterial organophosphate remediation. The proposed sequence treats chemical, molecular, microbiome, toxicological, and ecological evidence as complementary components of one validation chain and is summarized in Figure 2.

### 7.1. Site Characterization, Intervention Selection and Inoculant Design

Remediation strategy should begin with characterization of the contaminated matrix rather than selection of the strain showing the highest removal in culture. Site diagnosis should establish the identity, concentration, and spatial distribution of organophosphate residues and relevant transformation products, together with contamination history, soil physicochemical properties, residue accessibility, and the natural-attenuation baseline. Indigenous microbial mixtures recovered from chlorpyrifos-contaminated sites may retain useful transformation potential, but their presence alone does not establish complete pathway operation [88].

Biostimulation is appropriate when indigenous populations are present but constrained by measurable environmental factors such as moisture, nutrient availability, or aeration. Modelling-guided amendment selection can help identify such process limitations more specifically than nonspecific stimulation of microbial biomass [89]. Bioaugmentation is more defensible when indigenous transformation is absent, too slow, or terminates at a persistent product. Carrier-assisted systems can improve cell survival and spatial retention, but may also alter pesticide sorption and extractability; carrier-only, inoculum-only, combined, and untreated controls are therefore needed to separate biological and physicochemical contributions [50,90].

Consortium design requires additional functional resolution. Native soil microbiota can modify the persistence and interactions of introduced strains, while chlorpyrifos-transforming consortia may outperform individual isolates without revealing which member hydrolyses the parent compound, transforms TCP, or provides an indirect ecological function [91,92,93]. Functional complementarity should therefore be tested by comparing individual isolates, defined combinations, and the complete consortium under identical chemical conditions. Selective omission and reintroduction of individual members, combined with strain-specific qPCR and transcript, protein, or enzyme measurements, can determine whether particular populations are required for specific pathway steps or primarily support consortium stability.

### 7.2. Integrated Chemical, Molecular and Soil-Level Validation

Following intervention selection, the central analytical requirement is a continuous chemical sequence linking parent-compound depletion with formation and subsequent turnover of transformation products. Parent pesticide and diagnostic products should be quantified in parallel over time using abiotic, uninoculated, and treatment-specific controls. Analytical coverage must remain compound-specific: chlorpyrifos requires TCP monitoring; methyl parathion and parathion require PNP kinetics; diazinon requires IMP analysis; and malathion requires broader product screening because several ester-cleavage routes may operate.

Molecular evidence becomes most informative when synchronized with the measured chemical reaction. Multi-omics analysis, DNA stable-isotope probing, and compound-specific isotope analysis provide complementary approaches for linking environmental populations or molecular functions with pesticide-derived atoms and reaction timing [94,95,96]. Their interpretation should remain integrated with product-resolved chemistry because predicted genes may remain inactive and isotope incorporation can reflect downstream cross-feeding. Mechanistic attribution is strongest when residue kinetics, transformation-product profiles, strain-specific monitoring, and active gene, protein, or enzyme measurements are generated from the same experimental units.

Soil-level validation must then determine whether chemical transformation is accompanied by reduced biological risk and recovery of functions relevant to the intended land use. Compost, biochar, and bacterial inoculation can simultaneously alter pesticide availability, microbial activity, soil enzymes, and plant performance, making uncontaminated, contaminated, amendment-only, and biologically augmented controls important for causal interpretation [97,98]. Plant growth alone is insufficient as a detoxification endpoint because improvement may arise from nutrient mobilization, phytohormone production, or general stress alleviation independently of pesticide transformation.

Direct toxicity measurements provide stronger evidence of risk reduction. Bacterial inoculation, alginate encapsulation, vermiremediation, and combined earthworm–bacterial treatments have linked chlorpyrifos or profenofos dissipation with cytotoxic, genotoxic, faunal, or soil-fertility endpoints [99,100,101]. These systems also illustrate the need for factorial controls because carriers, earthworms, and amendments may alter aeration, aggregation, microbial dispersal, and pesticide accessibility independently of bacterial catalysis.

Integrated plant–microbe–carrier systems increase environmental realism but also complicate interpretation. A system combining an indigenous bacterium, *Ricinus communis*, and biochar accelerated chlorpyrifos removal and improved plant-performance indicators in soil microcosm and pot experiments [61]. Because the inoculant was identified as *Klebsiella pneumoniae*, however, demonstrated remediation performance does not remove the requirement for strain-level biosafety assessment before environmental release.

Experimental scale should therefore be reported explicitly rather than inferred from treatment complexity. Freshly spiked sterile soil, aged non-sterile soil, pot or mesocosm systems, and genuine field experiments represent distinct validation levels with different constraints on bioavailability, microbial competition, spatial heterogeneity, and treatment persistence. Functional persistence should be defined as maintenance of the required transformation activity throughout the remediation period rather than indefinite survival of the introduced population.

**Figure 3:**
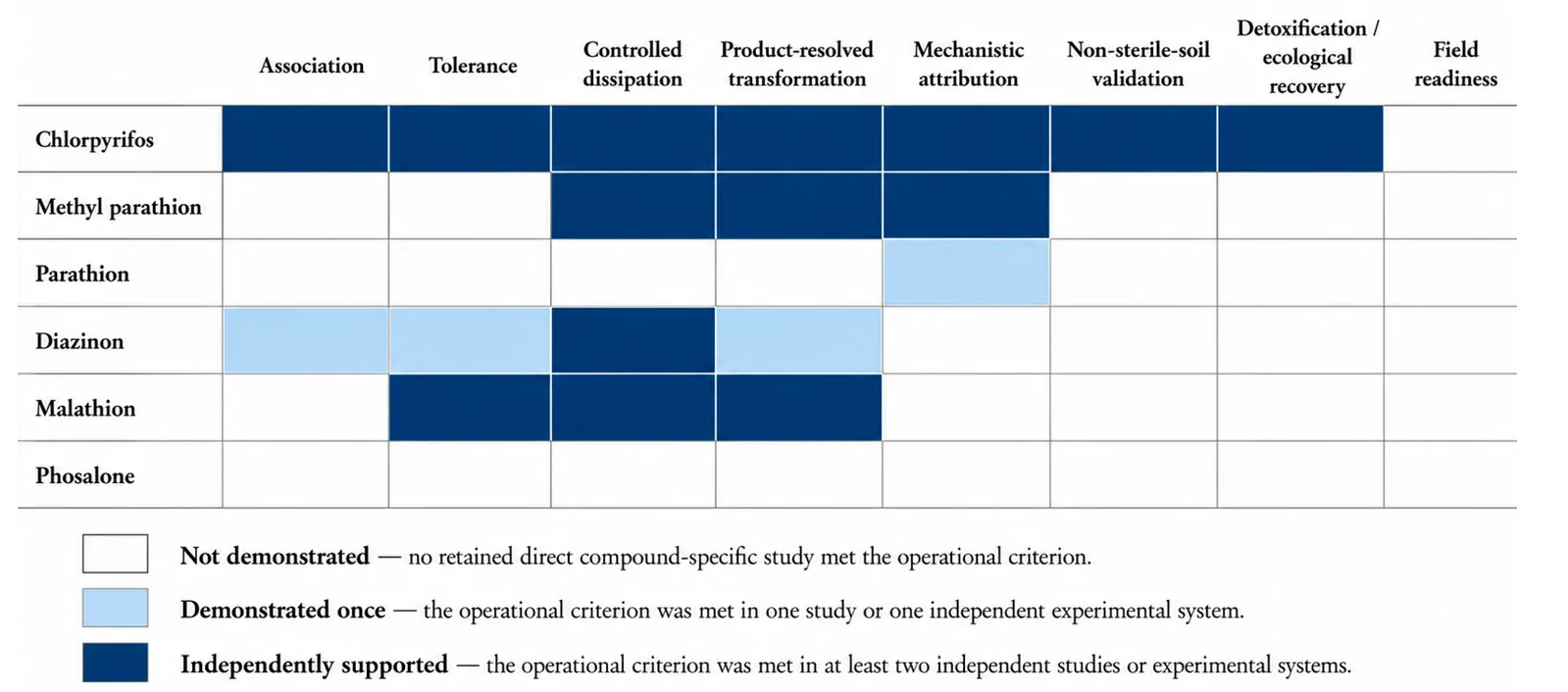
Evidence coverage across eight operational domains for the six focal organophosphate pesticides.

The strongest environmental evidence emerges when transformation-product turnover, active catalytic attribution, functional persistence, toxicity reduction, and recovery of a relevant soil or plant process converge within the same validation chain. Figure 3 summarizes the distribution and independent replication of evidence across the eight predefined domains for the six focal organophosphate pesticides, while Supplementary Table S2 provides the corresponding study-level assignments.

## 8. Future Research Priorities

Future progress in bacterial bioremediation of organophosphate pesticides requires a shift from isolate-centred screening towards continuous validation of chemical transformation, catalytic activity, and environmental recovery. Identification of tolerant bacteria remains useful for expanding biological resources, but newly isolated strains become mechanistically informative only when their activity is connected to transformation products, active catalytic functions, and reproducible performance under soil constraints. This need remains relevant for aged residues and historically contaminated agricultural sites, while emerging microbial-engineering approaches introduce additional questions concerning ecological persistence, genetic stability, and biosafety [5,55].

Future investigations should also extend beyond the small group of organophosphate pesticides that currently dominates the mechanistic literature. Comparative screening has demonstrated transformation potential against several organophosphate substrates, although tolerance and removal kinetics alone are insufficient to resolve complete compound-specific pathways [57]. More integrated evidence has been obtained for profenofos, for which *Cupriavidus nantongensis* X1ᵀ and the enzyme OpdB were linked to residue depletion, product formation, and reduced toxicity [102]. A separate consortium study resolved a multistep acephate-transformation route, illustrating how pathway analysis can be extended to structures that differ substantially from chlorpyrifos, parathion, or diazinon [103,104]. Comparable product-resolved studies are needed for dimethoate, phorate, fenitrothion, monocrotophos, phosmet, and other organophosphate pesticides for which bacterial evidence remains fragmented.

Where individual strains perform only part of a pathway, consortium development should be based on experimentally demonstrated reaction complementarity rather than taxonomic diversity or maximum total removal. The acephate-transforming consortium ZQ01 showed that successive reactions may be distributed across a mixed microbial system, although collective pathway performance does not identify the contribution of each member [103,104]. Plant–bacterial systems and constructed wetlands provide an additional route for integrating microbial transformation with contaminant uptake, rhizosphere processes, and hydraulic retention, as demonstrated in chlorpyrifos-removal experiments [105]. Their development requires factorial designs that separate plant, microbial, substrate, and physicochemical contributions and determine whether transformation products are eliminated rather than redistributed among environmental compartments.

Causal pathway reconstruction should accompany these ecological approaches. Candidate genes identified through genomes, metagenomes, or differential expression should be connected to substrate-specific protein activity and the expected chemical reaction through heterologous expression, purified-enzyme assays, targeted inhibition, gene disruption, or complementation. High-resolution mass spectrometry can reveal previously unrecognized products, but tentative assignments should not define a pathway until supported by diagnostic fragmentation, authentic standards, or orthogonal analytical evidence. Contemporary assessments of chlorpyrifos remediation and broader microbial-engineering approaches similarly emphasize the need to connect molecular observations with residue kinetics rather than interpreting genome annotation or predicted pathways as direct evidence of degradation [23,55].

Environmental validation should proceed through a planned increase in matrix complexity. Controlled soil systems can first identify matrix-related inhibition, whereas aged non-sterile soil is required to assess restricted bioavailability, natural attenuation, microbial competition, and inoculant persistence. Subsequent mesocosm, pot, constructed-wetland, and field experiments should retain compound-specific product analysis, strain or functional monitoring, and appropriate untreated, amendment-only, carrier-only, and inoculum-only controls. Current evidence indicates that chemical depletion, toxicity reduction, and ecosystem recovery remain inconsistently integrated, while plant–bacterial systems illustrate both the value and interpretive complexity of combining biological components [5,105].

Greater attention is also required for soils that remain underrepresented in bioremediation research, including saline, alkaline, water-limited, low-organic-matter, and chronically contaminated agricultural soils. Experimental conditions should reproduce realistic fluctuations in moisture and temperature, environmentally relevant residue concentrations, aged contamination, and co-occurrence of multiple pesticide classes rather than relying primarily on one freshly added compound at high concentration. Mixed-residue studies should retain separate analytical trajectories for each parent pesticide and its principal products because accelerated disappearance of one compound may coincide with inhibition, delayed transformation, or altered bioavailability of another. Physiological studies provide useful information on environmental tolerance, but require subsequent chemical and ecological validation in the target soil type [5,57].

Standardization of experimental reporting will be essential for comparing future studies and identifying systems suitable for scale-up. Reports should specify strain-level identity, inoculum density, pesticide formulation and purity, initial and time-resolved concentrations, soil properties, contamination history, residue ageing, extraction recovery, matrix effects, control treatments, product-identification confidence, and the statistical structure of biological replication. Whenever possible, inoculant persistence, catalytic-gene expression, toxicity endpoints, and functional recovery should be measured on the same temporal scale as residue and product kinetics. Such reporting would reduce inappropriate comparisons among high removal percentages obtained under fundamentally different experimental conditions.

The principal future objective is therefore to develop treatment systems whose mechanisms and environmental effects remain traceable as experimental complexity increases. Progress toward application requires reproducible performance beyond natural attenuation, turnover of relevant transformation products, persistence of the required catalytic functions, strain-level safety, and recovery of relevant soil or plant functions without additional ecological risk.

## 9. Conclusions

The six selected organophosphate pesticides occupy markedly different positions along the evidence continuum from initial transformation to environmental validation. Chlorpyrifos has the most developed parent–product evidence base, particularly for distinguishing primary hydrolysis from TCP turnover. Methyl parathion and parathion are comparatively well resolved at the hydrolase level, whereas diazinon and malathion retain important uncertainties in downstream-product fate. Phosalone remains the clearest evidence gap because the contemporary corpus does not yet establish an integrated strain–product–enzyme pathway. Translation of these findings into soil remediation requires convergence of compound-specific chemistry, catalytic attribution, environmental persistence, and biological recovery. Microbiome analyses can identify candidate populations and ecological responses, but reaction-specific causality depends on synchronized chemical and functional measurements. The field therefore needs to move beyond isolate catalogues and endpoint removal percentages toward reproducible validation chains that demonstrate transformation-product turnover, performance beyond natural attenuation, strain-level safety, and measurable recovery of relevant soil or plant functions.

## Data Availability

No new data were created or analyzed in this review. Data sharing is not applicable to this article.

## Supplementary Materials

The following supporting information can be downloaded at https://www.mdpi.com/article/10.3390/microorganisms14091974/s1: Table S1. Search strategy, eligibility criteria, and interpretive safeguards; Table S2. Compound-specific evidence maturity and principal validation gaps.
