# Supplementary material for: Metabolite-Resolved Validation of Bacterial Organophosphate Transformation in Soil: A Critical Review of Selected Organophosphate Case Studies
Source: Microorganisms. 2026 Sep 7;14(9):1974. doi: 10.3390/microorganisms14091974 (PMC13609906; doi:10.3390/microorganisms14091974)
Supplement: Supplementary file 1 [file microorganisms-14-01974-s001.zip › Table S1.pdf]

TABLE S1. SEARCH STRATEGY, ELIGIBILITY CRITERIA, AND INTERPRETIVE SAFEGUARDS

Search-date clarification. The original contemporary literature search covered publications through 30 June 2026. During revision, the database searches were rerun and updated on 26 August 2026. All retrieval, deduplication, and screening counts below refer to the updated search.

A. Database-specific search results

Database	Search/update date	Publication window	Search fields	Records retrieved
Scopus	26 August 2026	1 January 2020–26 August 2026	TITLE-ABS-KEY	682
PubMed	26 August 2026	1 January 2020–26 August 2026	Title/Abstract	254

B. Screening and evidence synthesis flow

Stage	n
Records retrieved from Scopus and PubMed	936
Duplicate records removed by DOI and bibliographic reconciliation	220
Unique records screened by title and abstract	716
Records excluded during title/abstract screening	434
Records shortlisted for detailed eligibility and evidentiary assessment	282
Database-derived publications retained in the evidence synthesis	41
Additional foundational, methodological, or contextual publications retained through citation tracking or targeted selection	40
Final evidence synthesis corpus	81

Reference-source separation. The final corpus comprised 41 database-derived publications and 40 additional foundational, methodological, or contextual publications, for a total of 81 publications.

C. Eligibility and exclusion criteria

Type	Operational criterion
Direct-evidence inclusion	Peer-reviewed primary study involving a bacterial strain, consortium, community, or bacterial catalytic system relevant to an organophosphate pesticide or a defined transformation product, with an experimentally interpretable chemical, molecular, toxicological, ecological, or environmental endpoint.
Comparator inclusion	Methodological or contextual publication retained to define analytical confidence, soil-matrix constraints, microbiome interpretation, isotope tracing, modelling, network analysis, or biosafety. Comparator evidence was explicitly labeled and did not contribute to compound-specific maturity scoring.
Date policy	The contemporary database window was 1 January 2020–26 August 2026. Pre-2020 publications could be retained only through citation tracking when they provided essential foundational evidence on pathways, transformation products, radiolabeled fate, or direct enzyme activity that could not be replaced by contemporary studies.

Type	Operational criterion
Exclusion	Duplicate records; studies unrelated to bacterial organophosphate transformation; studies of non-pesticidal organophosphate esters; clinical, host, or insect-gut studies; exclusively non-bacterial systems; predominantly physicochemical removal; analytical monitoring without relevant bacterial evidence; or insufficient experimental evidence for the claimed evidence domain.
Interpretive safeguard	Growth, optical density, taxonomic enrichment, predicted pathways, or endpoint parent-compound disappearance alone were not accepted as evidence of product-resolved transformation, mechanistic attribution, detoxification, or mineralization.

D. Reviewer involvement and disagreement resolution

Stage	Procedure
Primary screening and evidence extraction	L.A. performed the primary literature screening and evidence extraction using the predefined criteria.
Independent verification	D.E.Z. independently verified study eligibility and evidence-domain assignments.
Resolution of differences	Differences in eligibility or classification were discussed by L.A. and D.E.Z. and resolved by consensus. A.K.S. reviewed the final evidence classification.

E. Exact executed database search strings

Note. The search strings below are reproduced exactly as executed; wording and syntax have not been editorially altered.

Database	Exact executed query
Scopus	`TITLE-ABS-KEY(`<br>`(`<br>`"organophosphate pesticide*" OR "organophosphorus pesticide*" OR`<br>`"organophosphate insecticide*" OR chlorpyrifos OR "methyl parathion" OR`<br>`parathion OR diazinon OR malathion OR phosalone`<br>`)`<br>`AND`<br>`(bacter* OR microbial OR microorganism*)`<br>`AND`<br>`(biodegrad* OR biotransform* OR hydrolys* OR mineraliz* OR bioremediat* OR`<br>`metabolite* OR "transformation product*" OR pathway OR "catabolic gene*" OR`<br>`hydrolase* OR esterase* OR "enzyme activity")`<br>`)`<br>`AND PUBYEAR > 2019`<br>`AND PUBYEAR < 2027`
PubMed	`(`<br>`(organophosphate[Title/Abstract] AND pesticide*[Title/Abstract]) OR`<br>`(organophosphorus[Title/Abstract] AND pesticide*[Title/Abstract]) OR`<br>`(organophosphate[Title/Abstract] AND insecticide*[Title/Abstract]) OR`<br>`chlorpyrifos[Title/Abstract] OR "methyl parathion"[Title/Abstract] OR`<br>`parathion[Title/Abstract] OR diazinon[Title/Abstract] OR malathion[Title/Abstract] OR`<br>`phosalone[Title/Abstract])`<br>`AND`

Database	Exact executed query
	(bacter*[Title/Abstract] OR microbial[Title/Abstract] OR microorganism*[Title/Abstract])<br>AND<br>(biodegrad*[Title/Abstract] OR biotransform*[Title/Abstract] OR hydrolys*[Title/Abstract] OR mineraliz*[Title/Abstract] OR bioremediat*[Title/Abstract] OR metabolite*[Title/Abstract] OR "transformation product"[Title/Abstract] OR "transformation products"[Title/Abstract] OR pathway*[Title/Abstract] OR hydrolase*[Title/Abstract] OR esterase*[Title/Abstract] OR "enzyme activity"[Title/Abstract])<br>AND ("2020/01/01"[Date - Publication] : "2026/08/26"[Date - Publication])

F. Evidence-gap interpretation

Issue	Rule
Evidence maturity	Only evidence domains that met the operational criteria in Table S2 were assigned. Comparator publications were listed separately and were not used to increase compound-specific maturity.
Phosalone	Any conclusion concerning an unresolved phosalone pathway was restricted to the documented contemporary corpus and was not interpreted as evidence that bacterial phosalone transformation is biologically absent.

G. Reference-source categories (separated)

Reference-source category	n	Treatment in the review
Database-derived publications retained after screening	41	Included in the evidence synthesis after database retrieval and eligibility assessment.
Additional foundational, methodological, or contextual publications	40	Retained separately through citation tracking or targeted selection to support foundational pathways, methods, analytical interpretation, or context.
Total final evidence synthesis corpus	81	Combined corpus used for the review; comparator evidence remained separately labeled and did not increase compound-specific maturity scoring.
