# Supplementary material for: Metabolite-Resolved Validation of Bacterial Organophosphate Transformation in Soil: A Critical Review of Selected Organophosphate Case Studies
Source: Microorganisms. 2026 Sep 7;14(9):1974. doi: 10.3390/microorganisms14091974 (PMC13609906; doi:10.3390/microorganisms14091974)
Supplement: Supplementary file 1 [file microorganisms-14-01974-s001.zip › Table S2.pdf]

UPPLEMENTARY TABLE S2. COMPOUND-SPECIFIC EVIDENCE MATURITY AND PRINCIPAL VALIDATION GAPS

A. Operational definitions for the eight evidence domains

Domain	Minimum operational criterion	Evidence not sufficient
A – Association	Reproducible relationship between pesticide exposure and a bacterial taxon, community, abundance, network feature, or function.	Taxonomic enrichment or correlation alone does not demonstrate transformation.
T – Tolerance	Survival or growth under a defined pesticide exposure relative to an appropriate baseline/control.	Growth or OD increase does not demonstrate catalytic conversion.
D – Controlled dissipation	Time-resolved quantitative parent-compound loss interpreted against an abiotic, uninoculated, or equivalent control.	Single endpoint loss without an appropriate control is insufficient.
P – Product-resolved transformation	One or more transformation products detected in temporal and chemical association with parent-compound loss.	Predicted pathways without chemical product evidence are insufficient.
M – Mechanistic attribution	A gene, protein, enzyme, or catalytic function experimentally linked to the measured reaction.	Gene presence, annotation, docking, or proteomic association alone indicates mechanistic potential, not direct mechanistic attribution.
S – Non-sterile-soil validation	Relevant transformation phenotype demonstrated in non-sterile soil in the presence of resident microbiota and natural attenuation controls.	Liquid culture or sterile soil alone is insufficient.
E – Detoxification/ecological recovery	Directly measured reduction in toxicity or recovery of a defined soil/plant function after treatment.	Parent-compound disappearance alone is not detoxification.

F – Field readiness	Environmentally realistic field-scale efficacy together with persistence or functional maintenance, transformation-product control, and biosafety information.	Pot, greenhouse, microcosm, mesocosm, or constructed-wetland studies are not classified as field readiness.

B. Standardized experimental levels

**Code**	**Operational definition**
L1 – Defined liquid culture / enzyme system	Defined liquid culture, enrichment, purified/recombinant enzyme, or engineered cellular system.
L2 – Freshly spiked sterile soil	Sterile soil experimentally amended with the pesticide and evaluated under controlled laboratory conditions.
L3 – Freshly spiked non-sterile soil	Non-sterile soil experimentally amended with the pesticide, retaining resident microbiota and natural attenuation.
L4 – Aged or historically contaminated non-sterile soil	Non-sterile soil containing aged residues or contamination that predates the experimental intervention.
L5 – Pot / greenhouse experiment	Plant–soil experiment conducted in pots or greenhouse conditions.
L6 – Mesocosm / constructed wetland	Intermediate-scale contained environmental system, including biomixture modules or constructed wetlands.
L7 – True field validation	In situ field-scale intervention in environmentally realistic soil, with remediation/ecological performance assessed under field conditions.
NR-soil – Unresolved soil classification	A soil component was reported, but sterility, contamination age, or in situ status could not be resolved from the accessible report; no higher environmental level was inferred.

C. Analytical-confidence and enzyme-evidence coding

Code	Operational meaning
P0 – No product evidence	No transformation product was experimentally reported.
P1 – Indirect pathway assignment	A product or pathway was inferred from parent-compound disappearance, biochemical reasoning, prediction, or another indirect basis without direct analytical identification of the proposed product.
P2 – Tentative analytical identification	A product was assigned using a single analytical platform, retention behavior, molecular-ion information, or library matching without documented diagnostic fragmentation or authentic-standard confirmation.
P3 – Diagnostic analytical support	Product identity was supported by diagnostic fragmentation, MS/MS, accurate mass, or independent orthogonal analytical evidence, but authentic-standard confirmation was not documented.
P4 – Authentic-standard confirmation	Product identity was confirmed against an authentic analytical standard, including retention-time and/or spectral agreement.
G0 – No mechanistic evidence	No gene/enzyme evidence relevant to the transformation mechanism.
G1 – Candidate-level evidence	Gene detection, genome annotation, predicted enzyme, or other candidate-level evidence without functional causality.
G2 – Associated functional evidence	Expression, proteomic, or enzyme-activity evidence associated with transformation, but without a causal perturbation or purified/recombinant functional test.
G3 – Direct causal evidence	Purified/recombinant enzyme, catalytic-site mutation, knockout/complementation, or equivalent functional experiment directly linking a catalytic function to the reaction.

D1. Study-level experimental design and environmental scale

Study [Main-text Ref.]	Pesticide	Bacterial strain / consortium	Matrix / experimental scale	Experimental level	Pesticide concentration	Controls	Scale note

Farhan et al. (2021) [25]	Chlorpyrifos	*Bacillus cereus* Ct3	Laboratory soil; defined culture	L1 + NR-soil	75–125 mg/kg soil; resistance tested up to 125–175 mg/L	Uninoculated/experimental soil controls reported in source	Soil sterility/aging not explicit in accessible report.
Kumar et al. (2022) [26]	Chlorpyrifos	ERM C-1: *Pseudomonas putida* T7, *P. aeruginosa* M2, *Klebsiella pneumoniae* M6 + *Aspergillus terreus* TF1	Defined mineral-salt medium	L1	500 mg/L CP	Individual strains/consortium comparison; uninoculated control details NR in abstract	
Ambreen et al. (2021) [27]	Chlorpyrifos	*Bacillus thuringiensis* MB497	M-9 broth; soil slurry; soil microcosm	L1 + NR-soil	200 mg/L CP; 28 mg/L TCP	Matrix/incubation controls reported; exact composition NR in abstract	Soil microcosm present, but sterility/aging not explicit in accessible report.
Liu et al. (2023) [28]	Chlorpyrifos	*Bacillus cereus* H27	Defined culture / enzyme fractions	L1	NR in accessible abstract	Whole-cell vs intracellular/extracellular enzyme fractions; abiotic control details NR	

Yadav et al. (2024) [29]	Chlorpyrifos	Ten CP-tolerant isolates; strongest included *P. aeruginosa* Pa608, *P. monteilii* NBFPALD_RAS131, *S. maltophilia* PEG-390, *P. hibiscicola* R4-721	Defined culture	L1	50 mg/L CP; tolerance up to 1000 mg/L	Isolate comparisons; abiotic control details NR in abstract	
Castrejón-Godínez et al. (2022) [30]	Methyl parathion / PNP	*Burkholderia zhejiangensis* CEIB S4-3	Minimal salt medium	L1	50 mg/L methyl parathion	MP-present vs MP-absent proteomic treatments	
Zhao et al. (2021) [31]	Methyl parathion / PNP	Recombinant methyl-parathion hydrolase from *Azohydromonas australica* expressed in *E. coli*	Purified/recombinant enzyme system	L1	Exact assay concentration NR in abstract	Multiple OP substrates and metal/cofactor conditions	
Xu et al. (2022) [32]	Methyl parathion / PNP	Engineered *E. coli* BL-MP carrying opdS and pnpA–E	Engineered defined culture	L1	NR in abstract	Engineered strain vs pathway controls; exact design NR in abstract	
Xu et al. (2021) [33]	OPP / transformation-product context	Engineered *E. coli* BL-PNP	Engineered defined culture (PNP downstream metabolite)	L1	NR in accessible abstract	Engineered vs parental/vector controls described in source	

Haque et al. (2020) [34]	OPP / transformation-product context	*Leuconostoc mesenteroides* WCP307 genes opdA/opdE; recombinant *E. coli*	Fermentation-derived isolate / recombinant enzyme system	L1	Exact assay concentration NR	Wild-type/recombinant and catalytic-site mutant comparisons	
Wang et al. (2022) [35]	Diazinon	*Sphingobium* sp. DI-6	Defined culture	L1	100 mg/L diazinon	Uninoculated/medium control details NR in abstract	
Verma et al. (2021) [36]	Malathion	*Ochrobactrum* sp. M1D	Minimal salt medium	L1	100 mg/L malathion	Uninoculated control details NR in abstract	
Dar et al. (2022) [37]	Malathion	*Micrococcus* sp. MAGK3	Defined culture / optimization	L1	0.03% malathion	Optimization-factor comparisons; abiotic control details NR	
Ma et al. (2022) [38]	Malathion	*Pseudidiomarina homiensis* FG2; *Pseudidiomarina* sp. CB1 (among eight isolates)	Defined culture	L1	500 mg/L malathion	Strain comparisons; abiotic control details NR	
Cabrera et al. (2022) [46]	Chlorpyrifos	Indigenous soil microbiota; no defined inoculated bacterial strain	Non-sterile horticultural soil microcosms	L3	NR in accessible abstract	Three soil types including reference soil; untreated/amendment comparisons	
Nayak et al. (2021) [47]	Chlorpyrifos	*Ochrobactrum* sp. CPD-03	Sterile and non-sterile soil microcosms	L2 + L3	NR in abstract	Sterile vs non-sterile; bioaugmentation/biostimulation/carrier comparisons	

Salem et al. (2022) [48]	Chlorpyrifos	*Serratia rubidaea* ABS10	MSM; sterile and non-sterile soil	L1 + L2 + L3	25 mg/L in MSM; soil concentration NR	Sterile/non-sterile inoculated soils vs controls; 14C fate comparison	
Yadav et al. (2021) [49]	Chlorpyrifos	*Dyadobacter jiangsuensis* MTCC 12851 (formulated and free)	Aqueous medium; soil microcosm	L1 + NR-soil	NR in abstract	Formulated vs unformulated strain; soil controls	Soil microcosm present, but sterility/aging not explicit in accessible report.
Liu et al. (2024) [50]	Chlorpyrifos	Immobilized *Bacillus cereus* H27 on rice-husk biochar	Liquid medium; contaminated soil	L1 + NR-soil	25 mg/L in liquid; soil concentration NR	Immobilized vs free bacteria vs biochar groups	Contaminated-soil component reported; sterility/aging not explicit in accessible report.
Lu et al. (2020) [52]	Chlorpyrifos	*Pseudomonas stutzeri* A1501	Rice-vegetated soil; defined medium	L1 + NR-soil	1 and 8 mg/kg soil	With/without P. stutzeri and CP treatments	Rice-vegetated soil component reported; experimental soil level not fully resolvable

							from accessible report.
Lara-Moreno et al. (2022) [53]	Chlorpyrifos	*Bacillus megaterium* CCLP1; *Bacillus safensis* CCLP2	Liquid culture; non-sterile soil	L1 + L3	10 mg/L in liquid; soil concentration NR	Natural attenuation, bioaugmentation, biostimulation and RAMEB comparisons	
Adams et al. (2024) [56]	Chlorpyrifos	*Bacillus* sp. UPMB10	Defined culture	L1	4 g/L reported as optimized substrate concentration	Culture controls NR in abstract	
Lee et al. (2021) [57]	OPP / transformation-product context	*Lactobacillus plantarum* WCP931; recombinant *E. coli* carrying opdC	Defined/recombinant system	L1	NR in abstract	Wild-type/recombinant and catalytic-site mutant comparisons	
Aswathi et al. (2021) [58]	Chlorpyrifos	*Pseudomonas nitroreducens* AR-3	Defined culture / proteomics	L1	NR in abstract	CP-exposed vs control proteome	
Malla et al. (2023) [59]	OPP / transformation-product context	Consortium C3: *Lactobacillus plantarum*, *L. rhamnosus*, *Bacillus shackletonii*	Defined culture	L1	Optimized concentration; exact CP level NR	Optimization-factor comparisons	

Haque et al. (2022) [60]	Chlorpyrifos	*Enterobacter* sp. HSTU-ASh6	Defined culture; tomato field plant-growth test	L1	NR in abstract	Culture/plant controls reported; chemical control design NR	The field plant-growth component was not classified as field remediation validation.
Kiran et al. (2026) [61]	Chlorpyrifos	*Klebsiella pneumoniae* C78	MSM; soil microcosm; pot experiment	L1 + NR-soil + L5	200 mg/L in MSM; 100 and 200 mg/kg soil	With/without bacterial inoculation and biochar	Pot experiment is explicit; soil microcosm sterility/aging not fully resolvable from accessible report.
Hou et al. (2023) [63]	Chlorpyrifos	Native field soil microbiome	Wheat/maize agricultural field	L7	270/337.5 g a.i./ha recommended dose and double dose	Recommended vs double dose; untreated field control	
Cheng et al. (2023) [64]	Chlorpyrifos	Native field soil microbiome	Wheat/maize agricultural field	L7	Recommended and double	Recommended vs double dose and control plots	

					recommended rates		
Karpun et al. (2021) [65]	OPP / transformation-product context	Native orchard soil microbiota	Field orchard soil	L7	Commercial field doses of several pesticides	Field treatments vs baseline/control conditions	
Yadav et al. (2023) [71]	Chlorpyrifos	Native soil microbiome under aromatic grasses	Non-planted and planted non-sterile soil	L3	NR in abstract	Non-planted vs three planted treatments	
Singh et al. (2024) [72]	Chlorpyrifos	Native soil microbiome with biochar	Sandy-loam soil; plant-soil system	L3	2, 4, 6, 12 mg/kg CP; biochar 4%	With/without biochar; multiple CP concentrations	
Srinivasulu et al. (2024) [73]	Chlorpyrifos	*Pseudomonas citronellolis* CF3 plus native soil enzyme study	Defined culture; black/red agricultural soils	L1 + NR-soil	Soil pesticide doses 2.5–10 kg/ha; culture CP concentration NR	Dose series; soil comparisons; culture control NR	Agricultural-soil component reported; sterility/aging not explicit in accessible report.
Jeon et al. (2024) [82]	Diazinon	Diazinon-exposed soil community; 25 degrading isolates recovered	Greenhouse soil / isolate validation	L1 + L5	NR in abstract	Time-series exposed soil vs baseline/control	

Lian et al. (2022) [83]	Chlorpyrifos	MMI enrichment community; cultivable *Alcaligenes* sp. L1	Farmland soil enrichments / culture	L1	NR in abstract	MMI enrichment vs cultivable isolate comparison	
Wu et al. (2025) [87]	Chlorpyrifos	Microbial inoculant (composition NR in accessible abstract)	Non-sterile soil microcosm	L3	NR in abstract	CPF-only vs CPF + microbial inoculant	
Mansee et al. (2024) [88]	Chlorpyrifos	Six indigenous bacterial consortia isolated from biomixture modules	Biomixture modules / soil-like system; isolate consortia	L6	160 µg/g CP in three consecutive cycles	Treated vs untreated; stimulated vs non-stimulated mixtures	
Koippully Manikandan et al. (2024) [90]	Chlorpyrifos	*Aeromonas veronii* immobilized on rice-husk biochar (ARHB)	Water; non-sterile soil	L1 + L3	Pesticide concentration NR; ARHB 2 g/kg soil	ARHB vs bacterial/biochar treatments	
Islam et al. (2021) [92]	Chlorpyrifos	*Pseudomonas putida* CBF10-2 + *Ochrobactrum anthropi* FRAF13 + *Rhizobium radiobacter* GHKF11	Augmented and non-augmented agricultural soil	L3	NR in abstract	Native/non-augmented vs consortium-augmented soil	

Uniyal et al. (2021) [93]	Chlorpyrifos	ECO-M: *Agrobacterium tumefaciens* ECO1, *Cellulosimicrobium funkei* ECO2, *Shinella zoogloeoides* ECO3, *Bacillus aryabhattai* ECO4	Defined culture; contaminated-soil microcosm	L1 + NR-soil	50 mg/L CP in culture; real contaminated soil in microcosm	Consortium and soil microcosm controls reported	Contaminated-soil microcosm reported; sterility/aging not explicit in accessible report.
Saengsanga et al. (2023) [98]	Chlorpyrifos	*Priestia megaterium* NRRU-BW3; *Bacillus siamensis* NRRU-BW9; *Bacillus amyloliquefaciens* NRRU-TV11	Aqueous medium; CP-contaminated soil with rice seedlings	L1 + L5	NR in abstract	Uninoculated contaminated-soil controls	
Conde Avila et al. (2025) [99]	Chlorpyrifos	*Azotobacter vinelandii*, liquid or alginate-encapsulated	Agricultural soil	L3	250 mg/kg CP	Uninoculated soil; liquid vs encapsulated inoculum	
Gonzales-Condori et al. (2024) [101]	Chlorpyrifos + profenofos	Native bacterial consortium + *Eisenia foetida*	Experimental and real agricultural soils	L3 + L4	65.5 mg/kg CP + 112.95 mg/kg profenofos; real soil 0.801/0.592 mg/kg	Earthworm alone, consortium alone, combined and control groups	

Fang et al. (2023) [102]	Profenofos	*Cupriavidus nantongensis* X1^{T}; purified OpdB	Defined culture; purified-enzyme system	L1	20 mg/L profenofos	Whole-cell/purified-enzyme comparisons; expression analysis	
Lin et al. (2022) [103]	Acephate	Microbial consortium ZQ01	Defined MSM; contaminated soil bioaugmentation	L1 + L3	200 mg/L acephate in optimized culture; soil level NR	Consortium vs non-augmented soil; condition optimization	
Wu et al. (2023) [104]	Acephate	*Burkholderia* sp. A11	Defined culture; multiple non-sterile soils	L1 + L3	100 mg/L standard assay; tolerance 10–1600 mg/L; 50–200 mg/kg soil	Inoculated vs non-inoculated soils; condition optimization	
Aziz et al. (2022) [105]	Chlorpyrifos	*Acinetobacter baumannii* + *Bacillus cibi* with *Canna*/*Mentha*	Prototype constructed wetland	L6	NR in abstract	Vegetated wetland and bacterial–plant treatment comparisons	

D2. Study-level analytical confidence and evidence assignment

Study [Main-text Ref.]	Analytical method	Product-confirmation confidence (P0–P4)	Enzyme evidence (G0–G3)	Toxicity / functional endpoint	Assigned domain(s)	Evidence status
Farhan et al. (2021) [25]	Residual CP analysis; GC–MS metabolites; kinetic modelling	P3: TCP and DETP supported by retention time and diagnostic ions; authentic-standard	G0	No formal toxicity bioassay	T; D; P; S	Direct/compound-specific

		confirmation not reported.				
Kumar et al. (2022) [26]	HPLC; GC–MS; laccase activity	P2: Products/pathway reported by GC–MS; authentic-standard confirmation not reported.	G2: laccase activity associated with degradation	PGP traits only; no toxicity endpoint	T; D; P	Mixed microbial system; not used for bacterial-specific mechanistic attribution
Ambreen et al. (2021) [27]	HPLC parent/TCP; GC–MS metabolites	P2: GC–MS product assignments; authentic-standard confirmation not reported.	G0	None	T; D; P; S	Direct/compound-specific
Liu et al. (2023) [28]	HPLC–MS; DFT; enzyme-fraction assays; WGS	P2: HPLC–MS-supported product assignment; authentic-standard confirmation and diagnostic fragmentation were not explicitly documented.	G2: intracellular enzyme activity + genome candidates; no causal gene test	None	D; P; M	Direct/compound-specific
Yadav et al. (2024) [29]	Kinetic modelling; enzyme assays; dechlorination/CO2 measurements; pathway analysis	P1: Pathway assignment was reported, but direct product identification with diagnostic fragmentation or an authentic standard was not documented.	G2: enzyme activities associated with degradation	None	T; D; P	Direct/compound-specific

Castrejón-Godínez et al. (2022) [30]	HPLC for MP/PNP; 2D-PAGE; MALDI-TOF	P2: PNP/intermediate turnover monitored by HPLC; authentic-standard confirmation or diagnostic fragmentation was not explicitly documented.	G2: differential proteomics/pathway-enzyme expression; not causal in this study	None	D; P	Direct/compound-specific
Zhao et al. (2021) [31]	Recombinant protein purification; substrate-specific enzyme assays	P0: no product-resolved metabolite analysis reported	G3: cloned/expressed enzyme with substrate-specific catalytic activity	None	M	Direct/compound-specific
Xu et al. (2022) [32]	Chemical monitoring of MP/intermediates; synthetic pathway engineering	P2: Sequential intermediate turnover was experimentally monitored, but authentic-standard confirmation or diagnostic fragmentation was not explicitly documented.	G3: engineered multigene pathway provides causal attribution	None	D; P; M	Direct/compound-specific
Xu et al. (2021) [33]	Chemical monitoring of PNP to β-ketoadipate; pathway engineering	P2: Downstream product conversion was experimentally monitored, but authentic-standard confirmation or diagnostic	G3: engineered hydroquinone-pathway genes causally support conversion	None	P; M	Downstream transformation-product mechanistic evidence

		fragmentation was not explicitly documented.				
Haque et al. (2020) [34]	Recombinant enzyme assays; SDS-PAGE; catalytic-site mutation	P0: no product-resolved metabolite confirmation used for classification	G3: recombinant OpdA/OpdE + Ser→Ala loss-of-function	None	M	Mechanistic comparator; not soil-validation evidence
Wang et al. (2022) [35]	GC–MS metabolites; degradation kinetics	P2: Seven GC–MS metabolites; authentic-standard confirmation not reported.	G0	None	T; D; P	Direct/compound-specific
Verma et al. (2021) [36]	GC–MS metabolites	P2: GC–MS pathway metabolites; authentic-standard confirmation not reported.	G0	None	T; D; P	Direct/compound-specific
Dar et al. (2022) [37]	UHPLC; GC–MS; Taguchi DOE	P2: GC–MS fragments/pathway proposed; authentic-standard confirmation not reported.	G0	None	D; P	Direct/compound-specific
Ma et al. (2022) [38]	Metabolite analysis; carboxylesterase-gene inventory	P2: Mono-/dicarboxylic metabolites identified; authentic-standard confirmation not reported.	G1: carboxylesterase genes; no direct enzyme causality	None	T; D; P	Direct/compound-specific

Cabrera et al. (2022) [46]	Pesticide residue kinetics; seed-germination bioassay	P0: no product-resolved bacterial pathway	G0	Lettuce seed germination/elongation	A; S; E	Environmental comparator; no strain-specific bacterial attribution
Nayak et al. (2021) [47]	Time-resolved CP residue analysis; 16S rRNA and opd-gene monitoring	P0: no product-resolved evidence reported	G1: opd-gene monitoring; no causal enzyme test	None	D; S	Direct/compound-specific
Salem et al. (2022) [48]	14C-chlorpyrifos fate; parent dissipation	P0: No structurally resolved transformation product. The 14C experiment supports pesticide-derived carbon fate/mineralization and is evaluated separately from product-identification confidence.	G0	None	D; S	Direct/compound-specific
Yadav et al. (2021) [49]	Parent-residue analysis; metabolite detection	P2: TCP and other metabolites detected; authentic-standard confirmation not stated	G0	None	D; P; S	Direct/compound-specific
Liu et al. (2024) [50]	Parent-residue analysis; Biolog-ECO; T-RFLP; RT-PCR	P0: No transformation-product evidence reported.	G1: nitrogen-cycling genes monitored, not CP-catabolic causality	Soil microbial functional response	D; S; E	Direct/compound-specific

Lu et al. (2020) [52]	Nitrogenase activity; ROS; qPCR nifA/nifH	P0: No transformation-product evidence reported.	G2: nifA/nifH expression linked to toxicity response, not degradation	Nitrogen-fixation inhibition	A; E	Direct/compound-specific
Lara-Moreno et al. (2022) [53]	Parent-residue analysis; soil toxicity bioassay	P0: No transformation-product evidence reported.	G0	Final soil toxicity reduced to undetectable levels in inoculated treatments	D; S; E	Direct/compound-specific
Adams et al. (2024) [56]	GC–MS	P2: Metabolites assigned by GC–MS; authentic-standard confirmation not reported.	G0	None	D; P	Direct/compound-specific
Lee et al. (2021) [57]	Recombinant enzyme assay; mutation analysis	P0: parent depletion only	G3: opdC cloning + Ser116→Ala loss-of-function	None	D; M	Mechanistic comparator; food/culture system
Aswathi et al. (2021) [58]	Quantitative proteomics	P0: No transformation-product evidence reported.	G2: CP-responsive protein remodeling; no causal catalytic validation	None	D	Direct/compound-specific
Malla et al. (2023) [59]	GC–MS; genome sequencing; RSM	P2: GC–MS pathway proposal; authentic-standard confirmation not reported.	G1: genome-supported candidate functions	None	D; P	Direct/compound-specific

Haque et al. (2022) [60]	GC–MS/MS; WGS; in silico modelling	P3: TCP and diethyl methyl-monophosphate by tandem MS	G1: genome/in-silico candidate enzymes; no causal enzyme experiment	Tomato growth promotion; not a remediation toxicity endpoint	D; P	Direct/compound-specific
Kiran et al. (2026) [61]	Parent-residue analysis; 16S identification; plant-growth measurements	P0: No transformation-product evidence reported.	G0	Plant growth/PGP endpoints	D; S; E	Direct/compound-specific
Hou et al. (2023) [63]	Residue kinetics; 16S/community and functional-gene analyses	P0: No transformation-product evidence reported.	G1: functional-gene abundance (ecological response), not degradation causality	Soil C/N-cycle microbial response	A; E	Direct/compound-specific
Cheng et al. (2023) [64]	Residue kinetics; microbial abundance/community; functional genes; enzyme activities	P0: No transformation-product evidence reported.	G1: ecological functional-gene responses; not catabolic causality	Soil microbial/enzyme ecological effects	A; E	Direct/compound-specific
Karpun et al. (2021) [65]	Basal and substrate-induced microbial respiration	P0: No transformation-product evidence reported.	G0	Microbial respiration inhibition/recovery	A; E	Environmental comparator
Yadav et al. (2023) [71]	CP/TCP dissipation kinetics; soil-enzyme kinetics; microbial community	P2: TCP detected; authentic-standard confirmation not reported.	G0	Soil-enzyme functional response	A; P; S; E	Environmental validation; no strain-specific attribution

Singh et al. (2024) [72]	Residue kinetics; bacterial community; soil enzymes; plant uptake	P2: TCP observed; authentic-standard confirmation not stated	G1: predicted/functional genes; not reaction-specific causality	Plant growth/stress and soil-enzyme endpoints	A; P; S; E	Environmental comparator
Srinivasulu et al. (2024) [73]	Soil enzyme assays; OD; parent removal; 16S identification	P0: No transformation-product evidence reported.	G0	Soil enzyme activity	A; D; E	Direct/compound-specific
Jeon et al. (2024) [82]	HPLC; 16S rRNA profiling; PICRUSt2; isolate screening	P0: No transformation-product evidence reported.	G1: predicted xenobiotic genes; no causal enzyme evidence	Community diversity/function change	A; D	Direct/compound-specific
Lian et al. (2022) [83]	Magnetic isolation; HTS; product analysis; oph-gene detection	P2: TCP, DETP, TMP, and phosphorothioate products identified; authentic-standard confirmation not reported.	G1: ophB/ophC detection	None	A; D; P	Direct/compound-specific
Wu et al. (2025) [87]	High-throughput sequencing; soil chemistry and enzyme activities	P0: No transformation-product evidence reported.	G0	SOC/TN/AP and soil-enzyme recovery; network stability	A; E	Direct/compound-specific
Mansee et al. (2024) [88]	CP residue monitoring; dehydrogenase; microbial respiration	P0: No transformation-product evidence reported.	G0	Dehydrogenase and microbial respiration recovery	D; S; E	Direct/compound-specific
Koippully Manikandan	LC–MS products; parent removal;	P2: LC–MS products; authentic-standard	G0	Soil properties and plant growth	D; P; S; E	Direct/compound-specific

et al. (2024) [90]	soil/plant measurements	confirmation not reported.				
Islam et al. (2021) [92]	GC–MS kinetics and metabolites	P2: TCP, phosphorothioic acid, fumaric acid, ethanol by GC–MS	G0	None	D; P; S	Direct/compound-specific
Uniyal et al. (2021) [93]	GC–MS metabolites; factorial optimization; kinetics	P2: TCP and 2-hydroxypyridine by GC–MS	G0	None	D; P; S	Direct/compound-specific
Saengsanga et al. (2023) [98]	Parent degradation assay; plant-growth measurements	P0: No transformation-product evidence reported.	G0	Rice height, root length, biomass and vigour	D; S; E	Direct/compound-specific
Conde Avila et al. (2025) [99]	UHPLC parent analysis; Allium cytogenetics; Eisenia survival	P0: No transformation-product evidence reported.	G0	Cytotoxic/genotoxic damage; earthworm survival	D; S; E	Direct/compound-specific
Gonzales-Condori et al. (2024) [101]	HPLC parent/product analysis; avoidance/ecotoxicity tests	P2: TCP and 4-bromo-2-chlorophenol were reported by HPLC; authentic-standard confirmation or diagnostic fragmentation was not explicitly documented.	G0	Earthworm avoidance/ecological risk	D; P; S; E	Direct/compound-specific

Fang et al. (2023) [102]	Parent/product analysis; RT-qPCR; purified enzyme assay	P2: Profenofos/4-bromo-2-chlorophenol pathway; authentic-standard confirmation not reported.	G3: RT-qPCR + purified OpdB recapitulated degradation	Metabolite-toxicity analysis	D; P; M; E	Direct/compound-specific
Lin et al. (2022) [103]	HPLC parent kinetics; metabolite/pathway analysis	P2: Methamidophos, acetamide, and acetic acid; authentic-standard confirmation not reported.	G0	None	D; P; S	Direct/compound-specific
Wu et al. (2023) [104]	Parent degradation and metabolite analysis; 16S community profiling	P2: Six metabolites reported; authentic-standard confirmation not reported.	G0	Indigenous-community disturbance assessed; no toxicity bioassay	D; P; S	Direct/compound-specific
Aziz et al. (2022) [105]	GC–MS parent/metabolites over 12 weeks	P3: Metabolite ions/fragment information supported by GC–MS; pathway proposed.	G0	System performance; no formal toxicity endpoint	D; P; S; E	Mesocosm/constructed-wetland evidence; not true field readiness

E. Methodological and contextual comparator publications retained in the synthesis but excluded from compound-specific maturity scoring

Study [Main-text Ref.]	Provenance	Role in synthesis	Evidence assignment
Dhakal et al. (2025) [39]	Citation tracking / targeted contextual selection	Review/framework evidence for pesticide-biodegradation interpretation.	Comparator — not scored

O'Callaghan et al. (2022) [40]	Citation tracking / targeted contextual selection	Review of microbial inoculants; framework for inoculant performance.	Comparator — not scored
Li et al. (2024) [41]	Citation tracking / targeted contextual selection	Soil microscale heterogeneity/microhabitat methodology.	Comparator — not scored
Schwarz et al. (2022) [42]	Citation tracking / targeted contextual selection	Spatial pesticide-biodegradation modelling.	Comparator — not scored
Meftaul et al. (2020) [43]	Citation tracking / targeted contextual selection	Dimethoate sorption/desorption and soil-fate context.	Comparator — not scored
Karleuša et al. (2025) [44]	Citation tracking / targeted contextual selection	Dimethoate–clay interaction; matrix-availability context.	Comparator — not scored
Meftaul et al. (2022) [45]	Citation tracking / targeted contextual selection	Multi-pesticide soil fate and sorption context.	Comparator — not scored
Ding et al. (2024) [51]	Citation tracking / targeted contextual selection	Microplastic co-stressor effects on chlorpyrifos biodegradation; matrix context.	Comparator — not scored
Liu et al. (2022) [54]	Citation tracking / targeted contextual selection	Long-term inoculant legacy and ecological effects.	Comparator — not scored
Cycoń et al. (2026) [55]	Citation tracking / targeted contextual selection	Bioaugmentation framework/review.	Comparator — not scored
Bellotti et al. (2025) [62]	Citation tracking / targeted contextual selection	Genome-based biosafety assessment of microbial biostimulants.	Comparator — not scored
Streletskii et al. (2022) [66]	Citation tracking / targeted contextual selection	Review of pesticides and soil microbiota.	Comparator — not scored

Riedo et al. (2025) [67]	Citation tracking / targeted contextual selection	Pesticide effects on soil microbiome/ecological interpretation.	Comparator — not scored
Medo et al. (2021) [68]	Citation tracking / targeted contextual selection	Non-OP pesticide microbiome comparator.	Comparator — not scored
Swaine et al. (2025) [69]	Citation tracking / targeted contextual selection	Meta-analysis of pesticide effects on soil health.	Comparator — not scored
Khmelevtsova et al. (2024) [70]	Citation tracking / targeted contextual selection	Pesticide/fertilizer impacts on soil microbiome and functions.	Comparator — not scored
Kers et al. (2022) [74]	Citation tracking / targeted contextual selection	Microbiome diversity/statistical methodology.	Comparator — not scored
Khomich et al. (2021) [75]	Citation tracking / targeted contextual selection	Multivariate microbiome analysis methodology.	Comparator — not scored
Nearing et al. (2022) [76]	Citation tracking / targeted contextual selection	Differential-abundance analysis methodology.	Comparator — not scored
Djemiel et al. (2022) [77]	Citation tracking / targeted contextual selection	Taxonomy-to-function inference limitations.	Comparator — not scored
Toole et al. (2021) [78]	Citation tracking / targeted contextual selection	Functional prediction versus shotgun metagenomics.	Comparator — not scored
Wright et al. (2025) [79]	Citation tracking / targeted contextual selection	PICRUSt2 methodology/reference update.	Comparator — not scored
Malla et al. (2022) [80]	Citation tracking / targeted contextual selection	Metagenomic functional potential under persistent pesticide exposure.	Comparator — not scored

Maharana et al. (2024) [81]	Citation tracking / targeted contextual selection	Metagenomic response to prolonged pesticide exposure.	Comparator — not scored
Matchado et al. (2021) [84]	Citation tracking / targeted contextual selection	Microbial co-occurrence network methodology.	Comparator — not scored
Kishore et al. (2023) [85]	Citation tracking / targeted contextual selection	Co-occurrence network interpretation.	Comparator — not scored
Xun et al. (2021) [86]	Citation tracking / targeted contextual selection	Keystone-taxa/network stability methodology.	Comparator — not scored
Dhakar et al. (2022) [89]	Citation tracking / targeted contextual selection	Modelling-guided soil biodegradation intervention design.	Comparator — not scored
Velte et al. (2025) [91]	Citation tracking / targeted contextual selection	Synthetic-community/native-microbiome interaction methodology.	Comparator — not scored
Gautam et al. (2023) [94]	Citation tracking / targeted contextual selection	Non-OP multi-omics comparator.	Comparator — not scored
Hu et al. (2021) [95]	Citation tracking / targeted contextual selection	Stable-isotope/multi-omics comparator.	Comparator — not scored
Masbou et al. (2025) [96]	Citation tracking / targeted contextual selection	Profenofos environmental fate/CSIA; no bacterial reaction attribution.	Comparator — not scored
Aziz et al. (2021) [97]	Citation tracking / targeted contextual selection	Chlorpyrifos + amendment soil study; no strain-specific bacterial attribution.	Comparator — not scored
Tagliabue et al. (2025) [100]	Database search	Vermiremediation/soil-fertility endpoint comparator.	Comparator — not scored
